# Advancements in Green Nanoparticle Technology: Focusing on the Treatment of Clinical Phytopathogens

**DOI:** 10.3390/biom14091082

**Published:** 2024-08-28

**Authors:** Sunny Mukherjee, Anamika Verma, Lingxue Kong, Aravind Kumar Rengan, David Miles Cahill

**Affiliations:** 1 Department of Biomedical Engineering, Indian Institute of Technology Hyderabad, Kandi, Sangareddy 502284, Telangana, India; 2 Institute for Frontier Materials, Deakin University, Geelong, VIC 3216, Australia; 3 School of Life and Environmental Sciences, Deakin University, Waurn Ponds, VIC 3216, Australia

**Keywords:** infections, clinical phytopathogen, nanotechnology, anti-microbial, green chemistry

## Abstract

Opportunistic pathogenic microbial infections pose a significant danger to human health, which forces people to use riskier, more expensive, and less effective drugs compared to traditional treatments. These may be attributed to several factors, such as overusing antibiotics in medicine and lack of sanitization in hospital settings. In this context, researchers are looking for new options to combat this worrying condition and find a solution. Nanoparticles are currently being utilized in the pharmaceutical sector; however, there is a persistent worry regarding their potential danger to human health due to the usage of toxic chemicals, which makes the utilization of nanoparticles highly hazardous to eukaryotic cells. Multiple nanoparticle-based techniques are now being developed, offering essential understanding regarding the synthesis of components that play a crucial role in producing anti-microbial nanotherapeutic pharmaceuticals. In this regard, green nanoparticles are considered less hazardous than other forms, providing potential options for avoiding the extensive harm to the human microbiome that is prevalent with existing procedures. This review article aims to comprehensively assess the current state of knowledge on green nanoparticles related to antibiotic activity as well as their potential to assist antibiotics in treating opportunistic clinical phytopathogenic illnesses.

## 1. Introduction

Pathogenic microorganism–host interactions are multifaceted and may occur at any scale, ranging from the broad realm of the open field down to the molecule. Up until recently, the ability of phytopathogens to induce illnesses in humans was discounted. However, recent data reveal that infections induced by phytopathogenic bacteria, viruses and fungi may significantly affect human health and safety. The impact of phytopathogens on human health is a growing area of interest. Plant pathogens, including viruses, bacteria, and fungi, cause various diseases in plants. Recent research indicates that a number of these plant pathogens can also affect human health. Nazarov et al. (2020) [1] provide a comprehensive overview of the causes and mechanisms of infectious plant diseases caused by these pathogens. In the context of fungi, Jain et al. (2019) [2] note that pathogenic fungi lead to plant diseases such as anthracnose, leaf spot, rust, and blight, which can indirectly impact human health through impacts on agricultural productivity and food security.

While direct transmission of these diseases from plants to humans is rare, the broader impact on food supply and the potential for novel zoonotic diseases cannot be ignored. For instance, a deeper understanding of the environmental factors influencing plant fungal diseases, as explored by Jain et al. (2019) [2], is crucial in predicting and mitigating their impact on human populations. Therefore, continuing research in this field is essential for comprehensively understanding the intricate relationships between plant pathogens and human health. Evidence of infections induced via phytopathogens has surfaced lately despite the distinct evolutionary paths of mammalian and plant diseases. Despite being more often associated with causing illness in plants, several species in the *Burkholderia*, *Pantoea*, *Pseudomonas*, and *Rhizobium* genera may also infect humans. Understanding the scope of incidence and identifying disease prevention techniques will require further research into the relationships between plant pathogens and their human hosts at the molecular level.

Similarly, several bacteria often associated with mammalian hosts may also infect plants, including *Salmonella* [3,4], *Enterobacter* [5,6], *Shigella* [7], and *Enterococcus* [8]. Clinical isolates of phytopathogens, with a few notable exceptions, have primarily been collected from individuals who were either immunocompromised (due to illness) or had recently had surgery. Therefore, these phytopathogens are often seen as opportunistic microbes that may infect humans and animals. It is not surprising that no host-specific virulence factors have been discovered in clinical phytopathogens, given that these opportunists do not need them. 

Traditionally, pathogens have been divided into those that infect plants and the others that infect animals. Pathogens may infect hosts across the plant and animal worlds, but the current terminology does not do them justice. Bacteria that can infect humans and cause illness are classified as human pathogens under the present human-centric paradigm, even if they can infect other organisms in their environment. This review focuses on the phytopathogens which could infect humans, and the molecular mechanism of pathogenicity concerning phytopathogens and their human hosts. Here we also introduce nanosystems that may interact with and inhibit the growth of these phytopathogens.

**Figure 1:**
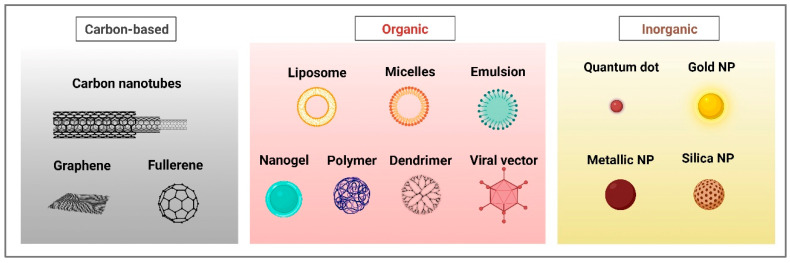
A broad classification of eco-friendly nanoparticles, highlighting carbon-based structures like nanotubes and graphene, organic assemblies including liposomes and dendrimers, and inorganic particles such as quantum dots and metallic nanoparticles, emphasizing the diversity of green nanotechnologies applicable in combating clinical phytopathogens.

New research into nanotechnology relies on elements found in nature. These natural sources include inorganic materials like ash, soot, and minerals as well as organics such as selenium and sulfur produced by many yeasts and bacteria or the cellulose found in cotton, hemp, wood, and different phytomaterials like propolis, which is a resinous exudate from different plant parts gathered and utilized by bees [9,10,11]. A comprehensive array of nanoparticles (NPs) are currently employed as drug delivery systems, encompassing carbon-based materials such as nanotubes and graphene, organic constructs including liposomes and dendrimers, as well as inorganic variants like quantum dots and gold nanoparticles, all of which represent the cutting edge of green nanotechnology with potential applications in the targeted treatment of clinical phytopathogens, as illustrated in Figure 1. 

Liposomes and lipid nanoparticles (NPs), other polymers, nanocrystals, polyethylene glycolated polymer (PEG) nanodrugs, metal and protein-based NPs, for example, are all commercially available nanostructures [12,13]. Synthesizing NPs by physical and chemical means, however, is both expensive and may be harmful to the environment. Therefore, to increase their use, it is necessary to create NPs using safe, eco-friendly processes. Making use of living things like plants and microbes is the most efficient method to do this [14]. The selection of incubation time, origin of the biological material, temperature and pH, are only a few of the crucial process factors pertaining to the biological synthesis of the nanoparticles [15,16]. In particular, green-based synthesis techniques may generate NPs with regulated dimensions. The pH contributes significantly in this regard [17,18]. Metal NPs may be biologically synthesized in a simple, eco-friendly and economical process [19]. This trend seems to be gaining momentum, with high expectations focused on nanoparticles made with the help of plants [17].

Interest in green nanotechnology is growing in the medical field because it can use naturally occurring resources like plants as “eco-friendly nano factories” and promotes further research into these plants to fulfil the growing requirement in a variety of industries [11,14,20]. This eco-friendly method utilizes biological agents, particularly plants, to perform the functions of decreasing and encapsulating chemicals. For instance, green chemistry-produced AgNPs provide a promising new option to conventionally synthesized NPs [21]. Due to their nano textured cell walls (silica-based), which can be altered in vivo and lower the cost of cell culture), plants become a rational choice for raw materials pertaining to industrial applications, as well as the production of green nanoparticles [11,22]. Biogenic NPs have been demonstrated to be more efficient against microbes than chemically manufactured NPs, both on their own and in combination with existing antibiotics. Additionally, biogenic NPs are low-cost to manufacture, non-toxic and safe for the surrounding environment [15]. 

Compared to the intracellular method, the extracellular route of bacterial production is preferred for recovering NPs that originated from microorganisms. That being said, supernatant, biomass, cell-free extract, and derivative components may all be used in the bacterial manufacture of intracellular and extracellular AgNPs [23,24]. AgNPs are synthesized with the help of a number of different molecules and biochemical processes, including peptides, c-type cytochromes, silver-resistant genes, reducing cofactors and a wide variety of enzymes. Cyanobacteria, seaweeds, and microalgae [25] are all often employed in more basic procedures to create NPs [19]. It has only been recently shown that the microbiome from the human gut is capable of biosynthesizing AgNPs as well [26]. An intriguing angle is that the potential impact of AgNPs on gut health may ultimately prove more successful than antibiotics [27]. However, secondary metabolites in plants have been proposed as the ideal candidates for biosynthesis of NPs on a large scale [28]. These metabolites provide an excellent stable environment and quicker production rate of the NPs than those in microbes [29,30]. 

Nanomaterials found in nature have been shown to have unique chemical and physical characteristics, to be biologically active, and to have intriguing medical and nutritional uses [11]. Presently, the trend of antibiotic misuse in medicine has resulted in the increasing emergence of antibiotic-resistant microorganisms on a global scale, causing a surge in the incidence rate of infections that have emerged as an alarming concern in the health sector due to their high mortality rates and their potential to become the next pandemic [28,31,32,33,34]. An effective and promising solution to drug-resistant opportunistic pathogens are NPs derived from plants, which may then be used to treat these pathogens and reduce the associated consequences of a wide variety of infectious illnesses [32]. Because of their great potential for sustainability and the invention of new antibiotic formulations with the promise of future medical exploration, this review focuses on the recent developments in green-synthesized anti-microbial NPs and the current methods established for synthesizing them.

## 2. Phytopathogenic Infection in Humans

### 2.1. Bacterial Phytopathogens

It has been documented that several bacterial species that cause disease in plants may also cause ailments in humans. For instance, it was discovered that some species of *Erwinia* and *Agrobacterium*, well-known phytopathogens that are capable of infecting a diverse selection of different plant hosts, may also induce opportunistic infections in human hosts [35]. A disease known as crown gall is developed in plants when the bacterium *Agrobacterium tumefaciens* colonizes the roots. In humans, *Agrobacterium tumefaciens* produced catheter-associated and non-catheter associated bacteremia in immunocompromised individuals and patients suffering from neutropenia [36]. When such infections caused by unclassified *Agrobacterium* were accounted, then an incidence rate of more than 20 infections was reported [37]. *Agrobacterium tumefaciens* was also shown to be the causative agent in a case of infectious keratitis that was acquired via the use of contact lenses [38]. 

Certain plant pods and seeds were discovered to be susceptible to infection by *Erwinia persinicus*, a phytopathogen, which was initially identified as the causal agent of disease in cucumbers, tomatoes, and bananas [39,40]. *E. persinicus* in humans was identified through a patient suffering from an infection of the urinary tract [41,42]. According to the study of Prod’homme et al. 2017 [43], *Erwinia billingiae* was shown to be the agent behind cutaneous infections that lead to human bacteremia. 

In addition to infections caused by species of *Erwinia* and *Agrobacterium*, various species of *Pantoea* and *Burkholderia* are also capable of infecting human hosts. In the 1980s, cystic fibrosis (CF) patients who had *B. cepacia* in their lungs died from a deadly pulmonary infection [44,45]. Three different strains of *B. cepacia* have been discovered to bypass barriers of the human respiratory tract and infect the epithelial cells. *Burkholderia gladioli* and *B. glumae* were discovered to induce pneumonia in chronic granulomatous disease patients, and *B. cenocepacia* caused septicemia in cystic fibrosis patients [46]. But the genetic differences prevailing between human and plant pathogenic strains of *Burkholderia* were not considered [47,48]. After incidents of catastrophic outbreak in Canada and the United States, some species of *Pantoea* previously recognized as plant infections only were reclassified as human pathogens. *Pantoea agglomerans* was responsible for 12 occurrences of infection in patients suffering from malignant tumors. These cases occurred in hospitals connected to a pharmacy sink that was contaminated by the utilization of infected intravenous items [49]. Patients with diabetes who had colonoscopies developed anal bleeding and a high temperature due to exposure to *Pantoea ananatis* [50,51]. Eight infants being cared for in neonatal critical care units developed sepsis due to a *Pantoea* infection caused by contaminated parenteral feeding in 2004. All infants passed away within six days of their diagnosis [52,53]. Despite this, the species of *Pantoea* that was involved could not be determined.

### 2.2. Fungal Phytopathogens

In humans, there have been several reports of infections caused by phytofungi, similar to those caused by phytobacterial pathogens. *Alternaria infectoria*, which is responsible for severe blossom blight, was connected to keratitis in post-traumatic infections and induced phaeohyphomycosis after kidney transplant [54]. Infections have also been induced by organisms belonging to the genus *Bipolaris*. *Bipolaris hawaiiensis* and *Bipolaris spicifera* were found infecting the surgical site after cardiothoracic surgery. *Bipolaris spicifera* is known to cause leaf spot disease on sugarcane and switchgrass as well as leaf blight on buffalo grass [55]. Similarly, *Bipolaris hawaiiensis,* known to cause necrotic lesions and chlorosis in bermudagrass, was found to infect a cardiothoracic surgery wound site [56]. It has been found that another species of the genus *Bipolaris*, *Bipolaris australiensis*, may cause ulcers in the cornea [57]. 

In addition to disorders brought on by mycotoxins, hospital reports showed that plant-associated fungi had the potential to cause direct human infection [58]. For instance, the fungus *Colletotrichum truncatum* and *Fusarium proliferatum* were extracted from clinical tissue samples at the infection site of the eye [59,60]. Different phytopathogens have been identified from infected human samples, and the genus *Cladosporium* contains many of them. These include *C. gustisporum*, *C. allicinum*, *C. flabelliforme*, *C. cladosporioides*, *C. halotolerans*, *C. ramotenellum*, *C. funiculosum*, and *C. macrocarpum*, [61]. In every instance, the presence of fungal infections was established using methods of cell culture and the analysis of intergenic rDNA spacer region sequencing data. Despite the expanding amount of information indicating that plant-associated fungi have the potential to infect people, the specific processes through which phytopathogenic fungi might induce illnesses in humans remains, for the most part, unclear.

### 2.3. Viral Phytopathogens

There is a widespread presence of plant viruses in the environment, and this presence includes their interaction with fruits and vegetables. Every day, people as well as livestock and other animal species are put in contact with these viruses, many of which are quite resilient. Viruses that infect plants and vertebrates are very specialized, and their range is limited to that of their specific host. There has been much research conducted on the transfer of viruses from animals to humans (for example, coronavirus) [62]. On the other hand, there is no evidence to suggest that viruses found in plants provide any kind of pathogenic risk to humans or any other animals; however, several resilient plant viruses have been shown to be capable of replicating within the bodies of other hosts like insects, which then act as transmission vectors. 

Although plant and animal viruses infect different kinds of animals and plants, genome-wide phylogenic analyses have shown that they may have tight genetic links that point to a similar ancestor [63,64]. Two stable plant viruses, the *Tobacco Mosaic Virus* (TMV) and the *Peppermild mottle virus* (PMMoV), have been discovered in human fecal samples. An early investigation of the viral metagenome in the stomach revealed an unexpected predominance of PMMoV, with 109 PMMoV virions found per gram of dry weight of feces matter. Further studies verified a positive association between the virion content of PMMoV and certain clinical symptoms, such as atypical pain and fever [27,63,65]. TMV is a very stable virus found in tobacco products and has been discovered in the bronchoalveolar lavage of intubated patients as well as in active and passive smokers’ lungs [66]. Patients infected with PMMoV displayed anti-PMMoV antibodies at a higher frequency than in blood samples from healthy controls [67,68]. In a similar vein, a greater titer of anti-TMV IgG was discovered in smokers when compared with non-smokers [69]. There are still many unanswered questions surrounding the ability of plant viruses to proliferate in the cells of animals and humans. Recent studies have discovered that TMV has the capability to invade human HeLa cells and depositing its viral proteins on the autophagosomal membranes [70]. 

In a similar vein, it has been observed that the *Cowpea mosaic virus* can attach to human endothelium cells via the surface vimentin protein [71,72]. In recent times, cowpeas, as well as two mammalian cell lines, have been infected with the insect virus known as the *Providence* virus [65]. It has been shown that the *Providence* virus may have the capability to replicate in vertebrate and plant cells, along with those of the insect host. There is an increasing amount of information that indicates the occurrence of plant viruses and their ability to replicate in other animal cells. This raises the prospect that plant viruses may be able to trigger illnesses in humans. Even though animal cells may be invaded by plant viruses, and the viruses can reproduce inside them, there has been no concrete indication that plant viruses have the capability to induce symptomatic illness in animals [65]. However, it is not possible to rule out the chance that plant viruses might cause sickness in animals, and it is important to take this risk into consideration. As we explore the intricate relationship between plant pathogens and the diseases they incite, it becomes paramount to understand the specifics of these interactions. The subsequent Table 1 offers a meticulous compilation of phytopathogenic agents, including bacteria, fungi, and viruses, detailing the symptoms they provoke in plants, their methods of transmission, and the associated health implications in humans. This collection not only provides a reference for the scientific community but also establishes the groundwork for exploring the efficacy of green nanoparticle technologies in mitigating these phytopathogenic threats.

**Table 1:** Comprehensive compilation of clinical phytopathogenic diseases caused by bacteria, fungi and viruses.

	Responsible Pathogen	Symptoms in Plant	Plant Vectors	Clinical Presentation	Reference
Virus	TMV	Yellowing, Stunting	Tobacco, Tomato	Pulmonary disease	[73]
	PMMoV	Chlorosis, Mottling	Pepper	Abdominal pain, fever	[65]
Bacteria	*Burkholderia cepacia*	Gladiolus corms	Onion	Septicemia	[74]
	*Burkholderia cenocepacia*		Maize		
	*Burkholderia glumae*		Tomato		
	*Burkholderia gladiol*		Rice		
	*Erwinia billingiae*, *Erwinia persinicus*	Fire blight	Rosaceae, Pear, Apple	Cervical lymphadenitis	[43]
	*Agrobacterium tumefaciens*	Crown gall disease	Eudicots	Bacteremia, Fetal death	[38,75]
	*Pantoea agglomerans*	Leaf spot blotches	Fruit bearing trees	Septicemia	[49]
	*Pantoea ananatis*				
Fungi	*Cladosporium angustisporum* *Cladosporiumallicinum* *Cladosporium flabelliforme* *Cladosporium ladosporioides* *Cladosporium funiculosum* *Cladosporium herbarum* *Cladosporium halotolerans* *Cladosporium perangustum* *Cladosporium macrocarpum* *Cladosporium tenuissimum* *Cladosporium ramotenellum* *Cladosporium subuliforme* *Cladosporium subinflatum* *Cladosporium sphaerospermum*	Blight, Leaf spot, Blossom	Echeveria, *Dendrobium*, Strawberry	Infection in the respiratory tract	[61,76]
	*Fusarium graminearum* *Fusarium proliferatum*	Root rot, Head blight	Tomato, Tobacco, Legumes, Cucurbits	Blood infection	[59]
	*Colletotrichum truncatum*	Blight, Lesion, Necrosis	Cereals, Citrus, Strawberry,	Ophthalmic infection	[60,77]
	*Alternaria infectoria*	Blight, Blossom	*Guayule*	Keratitis, Phaeohyphomycosis	[54,78]
	*Bipolaris australiensis* *Bipolaris hawaiiensis* *Bipolaris spicifera*	Necrotic lesions, Chlorosis, Leaf spot, Leaf blight	Switchgrass, Sugarcane, Bermudagrass, Buffalograss,	Corneal ulcer, Infection at surgical sites	[56,57]

### 2.4. Inoculation Requirements for Infectious Disease Transmission across Kingdoms

Animal and plant diseases have developed specific virulence factors to assist the effective infection of their primary hosts. These variables may be either genetic or environmental. Cross-kingdom infections are sporadic because of the fundamental differences in the physiologies of plants and animals. For cross-kingdom infections to take place, pathogens need to adapt to the unfamiliar environment of the non-primary host.

#### 2.4.1. Overcoming the External Physical Barrier

**Figure 2:**
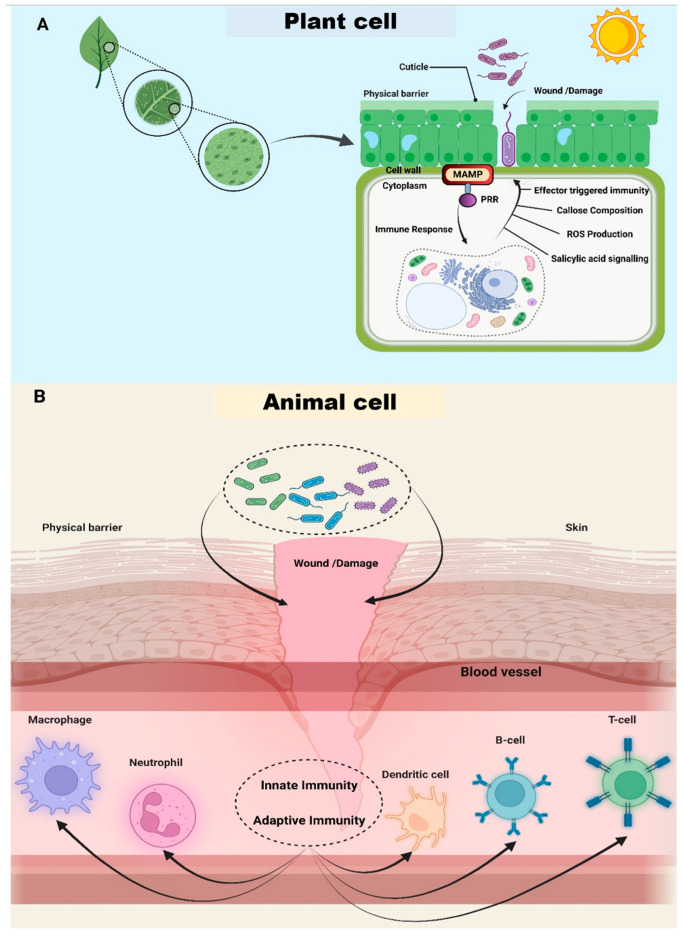
Plants and animals have unique defense mechanisms against pathogens. Physical barriers serve as plants’ and animals’ primary defense against potential pathogens. Although damage may make it possible for microorganisms to penetrate through physical barriers, additional preventative measures are used to fight infection. (**A**) Plants rely on processes of single-cell innate immunity. (**B**) Humans rely on host-specific immune cells, which are neutrophils, macrophages, B cells, T cells, and dendritic cells. Innate immunity is the primary barrier against foreign microbes. Microorganisms that cause diseases have developed unique mechanisms that allow them to subvert the immune systems of the organisms that serve as their main hosts, such as microbe-associated molecular patterns (MAMPs); effector-triggered immunity (ETI); reactive oxygen species (ROS); and pattern-recognition receptors (PRRs).

The pathogens that succeed in infecting plants or animals exhibit certain traits in common with one another. First, viruses must be able to endure the surrounding environment for an extended period before infection sites become available. Biofilms and chemotaxis are two strategies that bacteria might use to protect themselves from the damaging effects of their environment and gain access to the nutrients they need. In addition to having the ability to colonize host surfaces, infections need to break through the host’s physical defense barriers, such as the skin and mucin layer in animals and the epidermis, cuticle and cell wall in plants, or enter via wounds or other naturally occurring holes (Figure 2). Pathogenic microorganisms exhibit varied mechanisms to invade their hosts. In most scenarios, animal and plant pathogenic bacteria are unable to penetrate intact exterior defenses, such as skin or plant epidermis, without existing damage. This limitation necessitates their reliance on breaches in these barriers, including wounds or natural openings, to establish infections [79]. On the other hand, certain fungi possess the capability to actively permeate these external barriers, even when they are intact, thereby causing disease [80,81,82]. As a result, animal pathogenic bacteria typically invade hosts through openings caused by external factors, such as insect bites or medical interventions like catheter insertions [79]. In contrast to animal cells, plant cells are surrounded by a cell wall, which acts as an extra physical barrier. Bacteria that infect plants, such as species of the genus *Pectobacterium*, are capable of actively penetrating plant cell walls. They achieve this by releasing enzymes that degrade plant cell walls [83]. The localization of bacterial phytopathogens in host cells, whether in plants or animals, is host-dependent. Some animal pathogenic bacteria, such as *Listeria* [84], *Salmonella* [85], and *Mycoplasma* [86] species, can invade cells and proliferate there, while others are found only in the extracellular areas of animals [87]. A unique example of internal bacterial invasion of plant cells is the formation of bacteroid rhizobia within root nodules. However, this type of invasion is rare. Most bacterial phytopathogens thrive in the localized area of intercellular gaps of host plants, where they must evade the host plant’s defense mechanisms.

Animal viruses exhibit a range of mechanisms to infect hosts without the necessity of wounds. These viruses, differing fundamentally from bacteria, employ various transmission pathways. Notably, respiratory system transmission is a key route for viruses like Influenza and SARS-CoV-2, which spread through airborne droplets when an infected person coughs, sneezes, or talks, thereby attaching to receptors in the respiratory tract [88,89]. Additionally, digestive tract infection is common among viruses such as Norovirus and Rotavirus, transmitted typically through contaminated food or water, infecting the cells lining the intestine [90]. Sexual transmission is another critical pathway, especially for viruses like HIV [91] and HPV [92], which infect cells in the genital or oral mucosa during sexual activities. For instance, HIV, transmitted through sexual contact and blood, infects CD4+ T cells, leading to a gradual weakening of the immune system, while Influenza, a classic example of respiratory transmission, demonstrates its spread through airborne particles in crowded environments [88,93].

While both bacterial and viral pathogens have unique methods of infecting their hosts, key differences exist in their mechanisms. Bacteria, for instance, can actively degrade physical barriers like plant cell walls or exploit intercellular spaces, whereas viruses typically require an existing entry point or utilize alternative infection routes such as respiratory or digestive pathways. This distinction is crucial in understanding the challenges in developing effective treatments against these varied pathogenic strategies. The difference in their invasion tactics underlines the need for a diverse range of therapeutic approaches, including the potential use of green-synthesized antimicrobial nanoparticles. 

#### 2.4.2. Prevailing over the Host Immune System

The detection and subsequent reaction to xenogenic elements is a fundamental notion shared by the immune systems existing in plants [94] and animals [95]. The immune systems present in both animals and plants have receptors that may identify either particular or non-specific foreign pathogens. PRR proteins are the non-specific receptors that specifically recognize PAMPs or MAMPs, which are bacterial products. These molecular patterns trigger an immune response [96]. Nucleotide-binding oligomerization domains and leucine-rich proteins, or more particularly, R-proteins or resistance proteins, are the specialized receptors of plants that detect pathogen effectors within host cells. One of the most significant distinctions between plant and animal immunity is the activation of the adaptive immune system in animals under attack by foreign organisms or particles, which is absent in plants. However, innate immunity is used by cells in both animal and plant bodies [94,95].

Hence, plants do not have adaptive immune cells such as B and T cells that take part in the manufacture of antibodies. In addition, the innate immune systems of plants do not make use of circulating immune cells, in contrast to the immune systems of mammals. On the other hand, in contrast to mammals, plants can detect and react to microbial diseases at a single-cell level [97] (Figure 2A). 

Viruses, bacteria, and other infectious agents may be eliminated from an animal’s primary innate immune system by circulating phagocytes [98,99]. These phagocytes include macrophages, neutrophils, and dendritic cells (Figure 2B). The essential cellular function, phagocytosis, is responsible for engulfing and subsequently destroying invading pathogenic bacteria. During phagocytosis, phagosomes are recruited by the activation of vacuolar ATPase (v-ATPase) [100] for acidification of the phagosomal lumen in order to maintain a low pH environment favorable for optimal activity of the hydrolytic enzymes (such as lipases, glycosidases, proteases, and DNAses) [101,102]. Even though phagocytes are responsible for eliminating a wide variety of harmful bacteria present in the human host, many bacteria have evolved survival tactics to avoid being engulfed and destroyed while invading the host [103]. The human pathogens *Legionella pneumophila* and *M. tuberculosis* can live inside phagocytes by inhibiting the phagosomal lumen acidification process mediated by v-ATPase. This allows the bacteria to continue to thrive inside the phagocyte [104]. *Listeria monocytogenes* and *Pseudomonas aeruginosa* can withstand phagocytosis by manipulating the actin cytoskeleton and facilitating their exit from the phagosome [105].

In plants, receptors are responsible for the detection of PAMPs (such as peptidoglycans, lipopolysaccharides, bacterial flagellin, and chitin), which then stimulates various defensive responses. These responses include participation of mitogen-activated protein (MAP) kinases, ion fluxes and oxidative bursts. The next step is the systemic production of pathogenesis-related (PR) proteins, followed by the activation of different immune responses. Systemic acquired resistance (SAR) is another mechanism used by plants to generate secondary immune responses [106] in the regions of the non-infected distant tissues. Jasmonic and salicylic acid, both of which are well-known phytohormones, are responsible for mediating SAR. Using mobile signal transduction, these phytohormones can trigger long-distance immune activation in non-infected locations. At the location of the first infection, signaling molecules such as pipecolic acid [107], lipid transfer proteins, glycerol-3-phosphate, methyl salicylic acid [108], and azelaic acid [109] are generated. These signaling molecules are then transported to uninfected regions, activating SAR and preventing further infection [110].

On the other hand, several plant-associated bacteria and fungi have evolved resistance mechanisms that allow them to overcome the immune system of plants. Some plant pathogenic bacteria inhibit calcium signaling, an essential part of plant immune systems, using extracellular polysaccharides [111,112]. Additionally, many pathovars of *P. syringae* generate phytotoxins capable of overcoming stomatal defense. Additionally, in an evolutionary arms race between phytopathogenic bacteria and plants, bacterial type III secretion system (T3SS)-related effectors [113] limit the immune response in plants. By directly attaching to the short RNAs responsible for mediating the host’s response to viral infection, plant viruses can disrupt the signaling pathways used by the host to silence defense genes [114].

#### 2.4.3. Molecular Pathogenic Pathways Implicated in Cross-Kingdom Infections

The underlying molecular processes responsible for the infection of plant species by different phytopathogens have been thoroughly described. On the other hand, the molecular mechanisms responsible for infection of hosts from other kingdoms are not entirely known. The vast majority of published literature proving phytopathogenic cross-kingdom infections are reports that merely highlight the specific bacterial species that have been detected in connection with disease signs in an infected host that were not predicted. Only a few studies have looked at the molecular features of infection that may occur across kingdoms. For example, some would suggest that *A. tumefaciens* triggers disease in humans by a mechanism distinct from the process of tumorigenesis in plants [75,115]. During the infection of plants and humans, *Burkholderia pseudomallei* and *Burkholderia plantarii* use a variety of unique pathogenicity factors. *Burkholderia pseudomallei* and *Burkholderia plantarii* use T3SS-related effectors to protect them from their hosts’ immune responses and release various effector proteins that cause different disease symptoms. On the other hand, the pqsA-pqsE operon (caused by *B. pseudomallei*) and rhamnolipids (caused by *B. plantarii*) were shown to be disease virulence factors in humans. Some bacteria employ the same virulence factors in human and plant hosts, which may be dangerous. For instance, the T3SS system acts as a crucial virulence factor in the development of human arthritis and in the infection of plant tops by *P. agglomerans*. *P. aeruginosa* uses the same pathogenicity factors in *Arabidopsis thaliana* and mice. These factors include *toxA*, *plcS*, and *gacA* [116]. Recent research has demonstrated that human-infecting pathogen *Shigella* species can produce type III effector proteins that, in the process, interact with plant target proteins, and have a critical contribution in human disease pathogenesis [7].

#### 2.4.4. Disruption of Specific Animal Immune Responses by Phytopathogens

Recent research has offered insights into how bacterial pathogens escape the non-primary host immune system and might survive. Even though the molecular mechanisms governing the processes of infections across kingdoms are not fully known, the scientific literature has furnished some fundamental knowledge regarding the same. Bacteria can effectively colonize and infect hosts from different kingdoms because they use analogous processes to subvert the immune defenses of hosts. Actin is a cytoskeletal protein that is present in all eukaryotic cells, meaning that it may be found in mammalian cells as well as plant cells. Oligomerization of actin’s globular subunits results in the formation of microfilaments, which serve as parts of cellular structure and regulators of different cellular functions. Actin-associated proteins facilitate plant growth, breakdown, and reorganization of microtubules. Microtubules are responsible for various immune-related processes, including the transport of specific proteins like PR proteins [117]. The development of plant-infecting microorganisms in specific sites like intercellular space, which is reduced by toxic compounds, including phytoalexins and PR proteins produced by the actin-based microtubule complex network combination, can occur when an immune response is triggered by a virulent pathogen [118].

**Figure 3:**
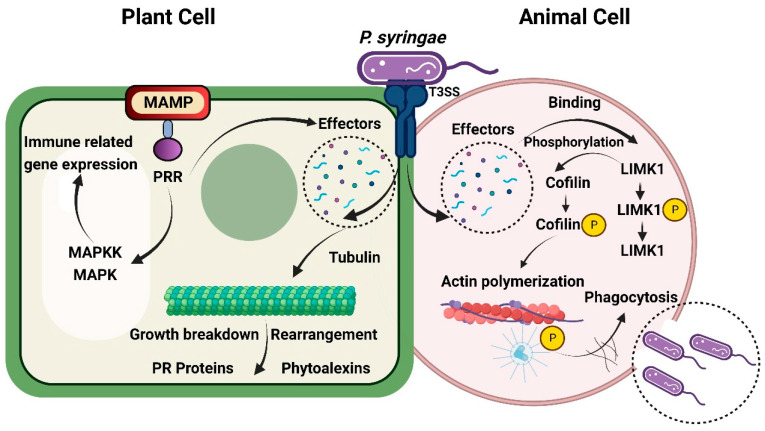
The effectors of phytopathogenic bacteria and their functions in the plants and animals they infect. Bacterial effectors provide particular functions in the process of defeating host defense mechanisms. Within the cells of plants, effectors are directed toward the host proteins, which perform a crucial role in the defense response in plants. The phytoalexin and PR protein generation process relies heavily on tubulin polarization, which specific effectors may inhibit when mounting an assault on the defense system. Phosphorylation of LIMK1, an enzyme that operates upstream of actin polarization and is targeted for phosphorylation by bacterial effector proteins in the macrophages of animals. The phosphorylation of LIMK1 leads to the phosphorylation of cofilin, which, in turn, impairs actin polarization and leads to an inability to phagocytose. PRRs: pattern-recognition receptors; LIMK: LIM kinase; MAPK: mitogen-activated protein kinase; PR protein: pathogenesis-related protein.

In mammals, the actin cytoskeleton is also involved in maintaining homeostasis in cells. The ability of macrophages to rebuild their actin is essential for their ability to move, phagocytose, and present antigens. For instance, phagocytosis relies almost entirely on actin rearrangement [119]. As a consequence, the majority of the bacterial pathogens that interfere with the actin regulatory system in plants can circumvent the immune responses of their animal hosts and vice versa (Figure 3). It was recently discovered that the plant pathogen *Pseudomonas syringae* uses similar tactics to evade the immunological response of animals and plants. The type III effector of *P. syringae* pv*. Tomato*, HopQ1, causes actin reorganization to be disrupted, which helps the pathogen escape immunological responses from macrophages in mice [120].

In addition, the effectors HopE1 and HopZ1a [121] specifically target tubulin and actin, which indicates that pathogenic bacteria may use disruption of the microtubule network as an efficient technique for bypassing human immunity [118]. In mouse models, HopQ1 facilitates invading bacteria’s phagocytosis by directly interacting with LIMK1 murine through cofilin1 phosphorylation. LIMK1 is a major component involved in the actin rearrangement [122]. Cofilin1 activity regulation plays a crucial part in the phagocytosis of bacteria during an infection by regulating the F-actin and actin cytoskeleton remodeling [123]. Similarly, published research has corroborated the function of bacterial effectors in selectively concentrating on the actin component responsible for the activation of immunity in plants [118]. Even while animal and plant cells both have the actin-based microtubule networks that play a crucial role in immune activation, there is not sufficient evidence to prove that phytopathogens specifically target actin signaling pathways in animals. Understanding the molecular pathways that underlie cross-kingdom pathogenicity will be facilitated by research demonstrating that common structures of the eukaryotic cell may be the target sites during the evasion of immune systems in animals and plants. 

## 3. Green Nanoparticles as Alternatives to Conventional Approaches to Disease Control

### 3.1. Introduction to Green Nanoparticles

Plant natural products are extracted from various plant tissues and organs. This process occurs due to plant secondary metabolism, which is under the influence of the surrounding environmental conditions and can be stimulated by external stimuli [124]. The excellent stability of plant-based nanoparticles has created interest in understanding and characterizing the processes of absorption and plant-based metal ion bioreduction. This interest was developed due to the biosynthesis of metal nanoparticles [125,126]. In this sense, a great number of research studies have demonstrated that plant extracts have the potential to serve as safe nanomaterial synthesis precursors. To this end, a myriad of secondary metabolites, namely saponins, tannins, steroids, flavonoids, and phenols [127] act as stabilizers in the process of bioreduction leading up to nanoparticle synthesis. The synthesis of various biobased nanoparticles, such as zinc oxide [128], copper [129], cobalt, platinum, magnetite, silver, and many others, has been successfully carried out using plant-based systems [19,130]. Live plants and plant extracts are another option for synthesis of nanoparticles [131]. When silver is present in the substrate, some plant species, such as *Brassica juncea*, *Medicago sativa*, or *Helianthus annuus*, can acquire a large quantity of silver in their tissues.

Interestingly, the NPs generated by plants come in a broader range of sizes and shapes than the nanoparticles manufactured via other mechanisms [126]. It has been discovered that the location of nanoparticle production has a profound influence on both the form and size of nanoparticles. Therefore, while obtaining NPs gathered in one plant, it is essential to remember that the others will differ depending on which portion of the plant is used for extraction [132]. Green-synthesized AgNPs were found to have an antifungal and antibacterial effect that was directly proportional to their concentration [133]. On the other hand, AgNPs synthesized from fungi were discovered to possess more effective antifungal activities against superficial mycoses, specifically *Malassezia furfur* and *Candida albicans* [21]. The formation of AgNPs from *Curcuma longa* was found to be in greater concentrations in the tuber extract than in the powder and was attributed to the easy availability of water-soluble reducing agents in the extract, primarily responsible for reducing silver ions in AgNPs [18,134]. It has also been reported that rapid synthesis of AgNPs can be accomplished in as little as 5 hours by reducing aqueous Ag^+^ ions with an extract of the tuber of the *Dioscorea bulbifera* [135,136] plant. The resulting AgNPs have potent antibacterial activity against both Gram-positive and Gram-negative bacterial strains. Because of its one-of-a-kind phytochemistry, this plant species also has significant applications in the medical field [137].

Plant-based AgNPs have received the most research attention because they are primarily connected with creating very effective antibacterial and antifungal capabilities (Table 2). Malabadi et al. [138] successfully synthesized AgNPs by using cell cultures derived from *Catharanthus roseus* and applying them as an aqueous extract. Their research was conducted in opposition to various clinical pathogens, such as *Escherichia coli*, *Bacillus subtilis*, *Candida albicans*, *Staphylococcus aureus*, and *Klebsiella pneumoniae*. It was shown that the stabilized AgNPs had the best efficiency possible against all the test pathogens. This rapid, economic, environmentally friendly, efficient method of NP production with specific and tunable properties provides optimism that biosynthesized NPs will be employed in the future [139]. 

Using the aqueous leaf extracts of *Crossopteryx febrifuga*, *Senna siamea* and *Brillantaisia owariensis*, it was possible to produce environmentally friendly AgNPs from these three plant species effectively. When contrasted with their respective crude extracts and AgNO_3_, the AgNPs that were produced from them showed a greater level of antibacterial activity against the three human skin bacteria that cause disease. This suggests that the biomolecules that coat the NPs have the potential to boost their biological activity [140]. A comprehensive list of green-synthesized nanoparticles with anti-microbial activity has been provided in Table 2.

**Table 2:** Green-synthesized nanoparticles with anti-microbial activity.

Plant Used	Part of Plant Used	NPs	Microorganism Used	Antibiotic Combined	References
*Curcuma longa*	Tuber extract	AgNPs	*Escherichia coli* BL-21	Not combined	[18,141]
*Dioscorea bulbifera*	Tuber extract	AgNPs	*Acinetobacter baumannii*	Piperacillin, erythromycin	[135,142]
			*Pseudomonas aeruginosa*	Chloramphenicol, vancomycin	
			*E. coli*	Streptomycin	
*Catharanthus roseus*	Cell cultures from roots, callus and leaves	AgNPs	*E. coli*, *Bacillus subtilis*, *Candida albicans*, *Staphylococcus aureus* and, *Klebsiella pneumoniae*	Not combined	[138,139]
*Senna siamea*, *Crossopteryx febrifuga* and *Brillantaisia owariensis*	Aqueous leaf extracts	AgNPs	Gram (+): *S. aureus* Gram (−): *E. coli*, *P. aeruginosa*	Not combined	[140]
*Fagonia indica*	Callus cell cultures	AgNPs	*E. coli*, *Salmonella typhi*, *Shigella sonnei*, and *Citrobacter amalonaticus*	Ciprofloxacin	[143]
*Urtica dioica*	Aqueous leaf extracts	AgNPs	Gram (−): *E. coli*, *Salmonella typhimurium*, *Serratia marcescens*, and *Klebsiella pneumoniae* Gram (+): *Staphylococcus epidermidis*, *Bacillus subtilis*, *Bacillus cereus* and *Staphylococcus aureus*	Ampicillin, amoxicillin, tetracycline, kanamycin, Amikacin, streptomycin, cefotaxime vancomycin, and cefepime	[79]
*Ocimum tenuiflorum*	Leaf extract	NiNPs	Gram (+): *Bacillus subtilis* and *Staphylococcus epidermidis* Fungi: *Aspergillus fumigatus*, *Aspergillus clavatus* and *Aspergillus niger Candida albicans* and *Candida tropicalis* Gram (−): *E. coli*, *Salmonella typhi* and *Klebsiella pneumoniae*	Not combined	[144]
*Piper guineense*	Aqueous leaf extracts	AuNPs	*S. aureus*, *Streptococcus pyogenes*	Not combined	[145]
*Zea mays*	Corn leaf waste extract	AgNPs	*S. typhimurium*, *S. aureus*, *E. coli*, *B. cereus*, and *Listeria monocytogenes*	Kanamycin, rifampicin	[146]
*Typha angustifolia*	Leaf extract	AgNPs	*E. coli* and *K. pneumoniae*	Gentamicin, cefotaxime, meropenem	[147]
*Phyllanthus reticulatus*, *Erigeron bonariensis*	Leaf extract	CuONPs	*E. coli*	Not combined	[148]

### 3.2. Green Nanoparticles: Detailed Overview, Mechanism of Action and Phytopathogenic Targets

Green nanoparticles are nanoscale particles synthesized using environmentally friendly methods that leverage biological entities such as plants, bacteria, fungi, and algae. These methods utilize natural reducing and stabilizing agents found in biological extracts, eliminating the need for toxic chemicals typically used in conventional nanoparticle synthesis [149]. The process involves mixing metal salt solutions with biological extracts, leading to the reduction of metal ions to nanoparticles, which are then stabilized by the organic compounds present in the extracts [150]. Green nanoparticles include metal nanoparticles like silver (AgNPs), gold (AuNPs), and zinc oxide (ZnO NPs); carbon-based nanoparticles such as carbon nanotubes and graphene; and organic nanoparticles like liposomes and dendrimers [151,152]. These nanoparticles exert their antimicrobial effects primarily through the generation of reactive oxygen species (ROS), membrane disruption, and biofilm inhibition on clinical phytopathogen targets including bacterial species such as *Burkholderia cepacia* and *Pantoea agglomerans*, fungal pathogens like *Alternaria infectoria* and *Fusarium proliferatum*, and plant viruses such as *Tobacco Mosaic Virus* (TMV) [153,154,155].

#### 3.2.1. Silver Nanoparticles (AgNPs)

Silver nanoparticles are among the most widely studied green nanoparticles due to their potent antimicrobial properties. For instance, AgNPs synthesized using *Azadirachta indica* (neem) leaf extract exhibit strong antibacterial activity against both Gram-positive and Gram-negative bacteria [156,157]. In another study, AgNPs synthesized using *Ficus benghalensis* bark extract demonstrated significant antifungal activity against *Candida albicans* and *Aspergillus niger*. The mechanism of action includes disruption of the bacterial cell membrane, generation of reactive oxygen species (ROS), and interference with DNA replication [158]. A study conducted by Tan et al. (2021) demonstrated that AgNPs synthesized using *Bacillus licheniformis* exhibited strong antibacterial activity against *Escherichia coli* (*E. coli*), *Bacillus cereus* (*B. cereus*), and *Ralstonia solanacearum* (*R. solanacearum*). This efficacy is attributed to mild acidity of AgNPs within bacterial cells, which leads to DNA and membrane damage, ultimately resulting in cell death, along with the ability of AgNPs to disrupt bacterial biofilms, enhancing antibiotic penetration and efficacy [159].

#### 3.2.2. Gold Nanoparticles (AuNPs)

Gold nanoparticles synthesized using plant extracts like Pelargonium graveolens have shown promising antimicrobial and anti-inflammatory properties. AuNPs are known for their ability to penetrate biofilms and disrupt microbial cell walls. In a study, AuNPs synthesized using *Terminalia arjuna* bark extract exhibited potent antibacterial activity against multidrug-resistant strains of *Pseudomonas aeruginosa* and *Klebsiella pneumoniae*. The mechanism of action includes binding to the bacterial cell wall and membrane, leading to structural damage and cell lysis [160]. Research by Arief et al. (2020) demonstrated that AuNPs synthesized using *Uncaria gambir* Roxb. leaf extract were effective in inhibiting the growth of *S. aureus* and *E. coli*. The study highlighted the potential of AuNPs in antimicrobial therapy, leveraging their ability to penetrate the biofilm and waxy cell wall of the bacterium and induce morphological distortions, leading to microbial cell death [161].

#### 3.2.3. Zinc Oxide Nanoparticles (ZnO NPs)

ZnO nanoparticles are known for their excellent antibacterial, antifungal, and wound-healing properties. These nanoparticles can be synthesized using extracts from *Aloe vera* [162], *Mussaenda* [163] and *Eucalyptus globulus* [164]. ZnO NPs exhibit a broad spectrum of antimicrobial activity by generating ROS, releasing Zn^2+^ ions that disrupt microbial enzymatic systems, and causing direct damage to microbial cell membranes. In a study, ZnO NPs synthesized using *Punica granatum* (pomegranate) [165] peel extract showed significant antibacterial activity against *Staphylococcus aureus*, *Enterobacter aerogenes*, *Pseudomonas aeruginosa*, and *Klebsiella pneumoniae*. A study by Gao et al. (2020) demonstrated that ZnO NPs synthesized using *Citrus sinensis* (orange) peel extract exhibited potent antifungal activity against *Botrytis cinerea*, a destructive phytopathogenic fungus [166]. The nanoparticles disrupted fungal cell walls and inhibited spore germination, highlighting their potential in agricultural disease management.

Green nanoparticles exhibit antibacterial activity through several mechanisms. They induce oxidative stress in bacterial cells by generating ROS such as superoxide radicals, hydroxyl radicals, and hydrogen peroxide. These ROS damage cellular components including lipids, proteins, and DNA, leading to cell death. For example, AgNPs generate ROS that oxidize thiol groups in enzymes and proteins, disrupting bacterial metabolic functions and causing apoptosis [167]. Nanoparticles also interact with the bacterial cell membrane, causing physical disruption and increased permeability, which leads to leakage of cellular contents and cell lysis. AgNPs, for instance, attach to the bacterial cell wall and penetrate the cell membrane, causing structural damage and disrupting the proton motive force essential for ATP synthesis [168]. Additionally, nanoparticles can bind to bacterial DNA, interfering with replication and transcription processes. This results in the inhibition of cell division and ultimately cell death. AuNPs have been shown to bind to the major grooves of bacterial DNA, blocking the access of replication enzymes and leading to bacterial cell death [169].

Green nanoparticles exhibit broad-spectrum antimicrobial activity, including antibacterial, antifungal, and antiviral effects. They penetrate the fungal cell wall and membrane, causing structural damage and leakage of intracellular components. ZnO NPs, for example, disrupt the chitin and glucan components of the fungal cell wall, leading to cell lysis and death [170]. Nanoparticles inhibit the germination of fungal spores, preventing the spread and colonization of fungi. AgNPs have been shown to inhibit the germination of Aspergillus spores by interfering with their metabolic processes and causing oxidative stress [171]. Furthermore, nanoparticles can inhibit viral replication by binding to viral surface proteins and preventing their attachment to host cells. They can also interfere with viral RNA and DNA synthesis. Studies have shown that AgNPs can inhibit the replication of viruses like HIV and influenza by interacting with viral glycoproteins and nucleic acids [172].

Biofilms are complex communities of microorganisms encased in a protective extracellular matrix, making them resistant to conventional antibiotics. Nanoparticles can penetrate and disrupt the biofilm matrix, exposing the embedded microbes to antimicrobial agents. AgNPs, for instance, disrupt the extracellular polymeric substances (EPS) matrix of biofilms, reducing their structural integrity and making the bacteria more susceptible to antibiotics [173,174]. Additionally, nanoparticles prevent the initial attachment and colonization of microbes on surfaces, inhibiting biofilm formation. ZnO NPs have been shown to inhibit the adhesion of *Pseudomonas aeruginosa* and *Staphylococcus aureus* to medical device surfaces, preventing biofilm development [165].

### 3.3. Synthesis of Nanoparticles

#### 3.3.1. Influence of Different Physicochemical Characteristics on Nanoparticle Synthesis

**Figure 4:**
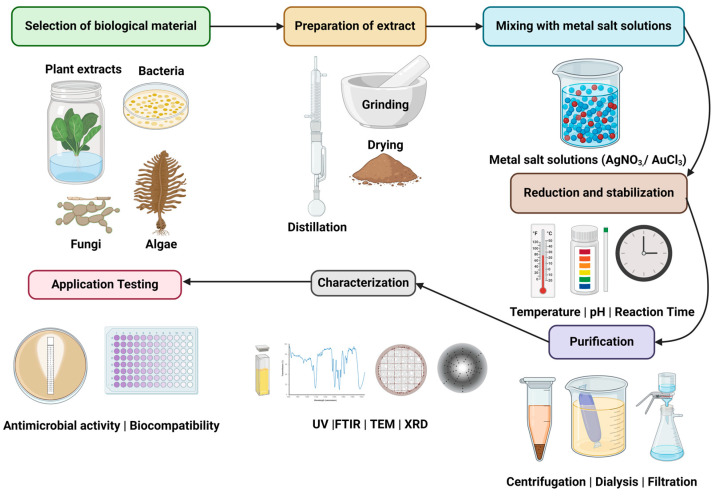
The diagram outlines the biosynthesis process for metal nanoparticles using biological materials. Starting with the selection of plant extracts, bacteria, fungi, or algae, the materials are processed into extracts. These extracts are mixed with metal salt solutions, facilitating the reduction and stabilization of nanoparticles under specific conditions. The nanoparticles are then purified and characterized using various techniques before undergoing biomedical application testing for antimicrobial activity and biocompatibility.

Several factors govern the nucleation and production of stable NPs. Crystalline NPs of varying forms and regulated sizes have been touted for a vast range of purported applications, such as anticancer, antioxidant, antibiofilm, antimicrobial and larvicidal effects. These characteristics are primarily dependent on the process constraints of the extract, as well as the response of the metal salt, pH, reaction duration, temperature, and metal salt concentration relative to plant extract [175]. The following sections provide a more in-depth discussion of each of these components during the growth period of the organisms (Figure 4). 

Several researchers have documented experimental attempts to optimize and improve the synthesis of NPs. In 2008, Kalishwaralal et al. explored nanoparticle synthesis under the influence of the growth phase of biomass [176,177]. The organism *Bacillus* sp. was shown to generate a comparatively large quantity of NPs during the stationary phase compared with the biomass derived from other phases. In the stationary phase of bacterial development, it was reported that NPs are packed densely within *E. coli* [178,179]. In the stationary phase, fungi secrete enzymes and other chemical compounds that increase their tolerance to stress, as reported in the scientific literature [180]. It has also been noted that different microbes and drugs have different tolerance capacities. For instance, *Aspergillus* sp. exhibits a longer mid-log phase due to the existence of nickel in the growth medium. However, evidence suggests that the inclusion of chromium in the medium would lengthen the stationary phase for the same organism [181,182]. However, the majority of published research suggests that stationary-phase microorganisms are optimal for NP production.

#### 3.3.2. pH and Precursor Concentration

Some researchers have shown that the molar ratios of reactants are also crucial criteria in chemical synthesis methods, with respect to determining the final NP size. It is well-known that the results of chemical synthesis may be affected by the concentration of the reactants. Perumal et al. showed that by manipulating the reactant concentration, the form of silver nanocrystals biologically synthesized from citrus leaf extract and silver nitrate can be methodically regulated [183]. It was reported that spherical NPs may be made using a 1:4 (*v*/*v*) ratio of AgNO_3_ to citric acid. However, it was also found that AgNP particle size was enhanced during bioorganic generation from plant extract. Precursors at a greater molar ratio had a substantial influence on NP structure, but no definitive association between precursor concentrations and nanocrystal shape was discovered. Additionally, it was mentioned that the reduction process for the metallic ions is very sensitive to the pH. Pandian examined how different pH levels influenced the production of CdS nanocrystallites by *Brevibacterium* species [144,184].

Studies have shown that protein-associated NPs may be more easily synthesized in an alkaline environment. As the pH of nanocrystallites was lowered from 7 to 6, particle agglomeration was observed [147,185]. The amount of AgNPs produced from *Cinnamomum zeylanicum* bark extract was shown to rise both with escalating percentage of bark extract and with increasing pH values in a separate investigation (pH ≥ 5) [186]. 

Nanocrystals also become precipitated out of solution at pH levels below 6. Methods of biomanufacturing metallic nanomaterials that have programmable functions and ordered nanostructures are essential for both basic research and practical applications because of their reduced toxicity, lesser creation of pollutants, and energy conservation. To create nanocrystals of varying sizes and morphologies, metal ions may be mineralized and reduced by microorganisms acting as efficient biofactories. Enhanced biosynthesis of nanoparticles increases production of these materials in a way that is both safe and sustainable [165].

#### 3.3.3. Temperature

Research shows that temperature has a significant impact on nanocrystal shape and dispersion. In most cases, researchers found that NPs shrank in size when subjected to higher temperatures. Increases in reaction temperature, from 25 to 60° Celsius, were shown to reduce the size of biosynthesized AgNPs from 35 to 10 nanometers [187]. Extract from sweet orange peel was used to kickstart the biosynthesis. With an elevation in reaction temperature, both reaction rate and particle formation rate increased. While the average particle size did decrease, the particle change rate escalated when the temperature was raised. In this regard, the temperature tolerance profile of the biological entity involved in NP synthesis is also crucial. Studies have shown that microorganisms may produce heat shock proteins in response to high temperatures, which aids in the creation of NPs.

## 4. Antibiotic Resistance and Phytopathogens in Therapeutic Settings

Antibiotic resistance has emerged as a critical global health issue, complicating the treatment of bacterial infections and increasing the morbidity and mortality associated with infectious diseases. The overuse and misuse of antibiotics in both clinical and agricultural settings have accelerated the evolution of resistant strains. Pathogens such as *Staphylococcus aureus*, *Pseudomonas aeruginosa*, and *Escherichia coli* have developed mechanisms to evade the effects of commonly used antibiotics, including the production of β-lactamases, modification of target sites, efflux pumps, and biofilm formation [188].

For instance, *Staphylococcus aureus* can produce β-lactamases that hydrolyze β-lactam antibiotics, rendering them ineffective. Methicillin-resistant *Staphylococcus aureus* (MRSA) strains carry the mecA gene, which encodes a penicillin-binding protein (PBP2a) with low affinity for β-lactams, thereby conferring resistance to methicillin and related antibiotics [189]. Similarly, *Pseudomonas aeruginosa* utilizes efflux pumps such as MexAB-OprM to expel antibiotics like ciprofloxacin and imipenem out of the cell, reducing their intracellular concentrations and effectiveness [190]. Phytopathogens, including bacterial species like *Burkholderia cepacia* and *Pantoea agglomerans*, fungal pathogens such as *Alternaria infectoria* and *Fusarium proliferatum*, and plant viruses like Tobacco Mosaic Virus (TMV), present significant challenges in therapeutic settings. These pathogens can cause severe infections in immunocompromised patients and complicate the management of chronic conditions. For example, *Burkholderia cepacia* is known for its intrinsic resistance to multiple antibiotics, complicating the treatment of respiratory infections in cystic fibrosis patients [191].

The resistance of phytopathogens to conventional antimicrobials underscores the need for innovative approaches to disease control. Research has shown that *Klebsiella pneumoniae* [192] and *Fusarium proliferatum* [193] can resist common antifungal treatments due to the presence of efflux pumps and the ability to form biofilms, which protect the fungal cells from antifungal agents.

While conventional nanoparticles have shown promise in various biomedical applications, their synthesis often involves the use of toxic chemicals such as sodium borohydride, hydrazine, and organic solvents. These harmful compounds pose several risks, including cytotoxicity, environmental pollution, and the generation of hazardous waste [194]. The accumulation of toxic by-products can lead to adverse effects on human health and the environment, limiting the practical application of these nanoparticles. Chemically synthesized silver nanoparticles (AgNPs), for example, have demonstrated excellent antimicrobial properties [195]. However, their production typically involves reducing agents like sodium borohydride, which can cause significant environmental damage and potential toxicity to human cells [196]. Another study highlighted the cytotoxic effects of chemically synthesized gold nanoparticles (AuNPs) on human lung fibroblast cells, emphasizing the need for safer synthesis methods [197].

Green nanoparticles offer a viable alternative to conventional nanoparticles by utilizing biological entities such as plant extracts, bacteria, fungi, and algae for their synthesis. This eco-friendly approach eliminates the need for toxic chemicals, reducing the environmental footprint and enhancing biocompatibility. The natural reducing and stabilizing agents in biological extracts ensure that the nanoparticles are both effective and safe for therapeutic use [198]. Green nanoparticles, therefore, represent a promising solution to the dual challenges of antibiotic resistance and the adverse effects associated with conventional nanoparticle synthesis.

Recent studies have demonstrated the potential of green nanoparticles in antimicrobial applications. For instance, silver nanoparticles synthesized using the leaf extract of *Azadirachta indica* (Neem) exhibited strong antibacterial activity against both Gram-positive and Gram-negative bacteria, with minimal cytotoxic effects on human cells [199]. Another study showed that gold nanoparticles synthesized using *Pelargonium graveolens* [200] leaf extract effectively inhibited the growth of multidrug-resistant *Streptococcus mutans* and *Candida albicans*, highlighting their potential in addressing antibiotic resistance.

## 5. Anti-Microbial Action of Nanoparticles

For bacteria to stay permanently attached, they must affix themselves to the surface and produce chemicals, such as proteins [201]. After they have established themselves, the bacteria begin to build colonies inside the peptidoglycan envelopes, ultimately forming a biofilm [202]. When this occurs, antibacterial agents cannot kill the germs, and the immune system cannot defend itself against the bacterium [203]. Additionally, biofilms have a poor response to antibiotics, which results in resistance to antibiotics [204].

Green nanoparticles exhibit potent antimicrobial effects due to several specific molecular interactions with microbial cells. These interactions disrupt various cellular processes, leading to the inhibition of microbial growth and cell death. The antimicrobial mechanisms of green nanoparticles are primarily driven by their ability to generate reactive oxygen species (ROS), interact with microbial cell membranes, and cause intracellular disruptions. These mechanisms collectively result in the observed antimicrobial effects. For instance, the generation of ROS such as hydrogen peroxide and hydroxyl radicals causes oxidative damage to cellular components, including lipids, proteins, and DNA, leading to cell death. Additionally, green nanoparticles interact with microbial cell membranes through electrostatic attraction, leading to membrane disruption and increased permeability, which causes leakage of cellular contents and further compromises cell viability. Intracellularly, nanoparticles interfere with essential metabolic processes by binding to enzymes and nucleic acids, thereby inhibiting replication and transcription, ultimately leading to cell death [205].

Studies have demonstrated that green nanoparticles synthesized from plant extracts like *Azadirachta indica* exhibit strong antibacterial activity against both Gram-positive and Gram-negative bacteria due to these combined mechanisms [206]. The linkage between these mechanisms and the observed antimicrobial effects is evident in the significant reduction in microbial viability and biofilm formation when treated with green nanoparticles. For example, gold nanoparticles synthesized using *Pelargonium graveolens* have been shown to penetrate biofilms and disrupt microbial cell walls, leading to structural damage and cell lysis [200]. Similarly, silver nanoparticles synthesized from *Aloe vera* extracts [207] and *Ginkgo biloba* leaf extract [208] generate ROS that oxidize thiol groups in microbial proteins, disrupting metabolic functions and causing apoptosis. These interactions illustrate the comprehensive antimicrobial action of green nanoparticles.

The key mechanisms include the generation of reactive oxygen species (ROS), interaction with microbial cell membranes, and interference with intracellular components [33].

### 5.1. Generation of Reactive Oxygen Species (ROS)

**Figure 5:**
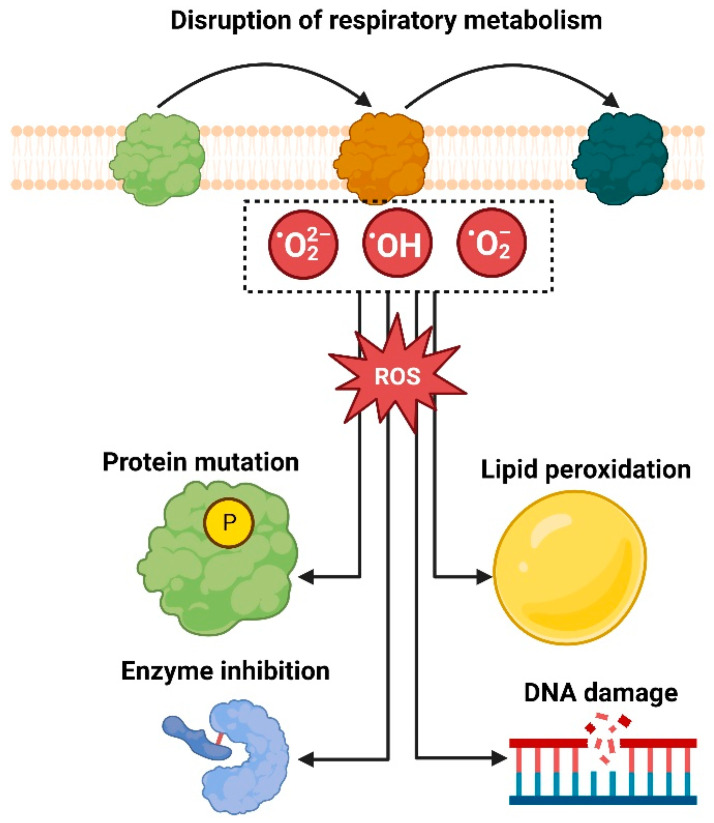
Anti-microbial action of nanoparticles by ROS generation and membrane disruption: The pathogenic impact of nanoparticles on cellular function, where disruption of respiratory metabolism leads to the generation of reactive oxygen species (ROS) (superoxide anion (O^2•−^), the hydroxyl radical (•OH), and another superoxide anion (O^2•−^)), which in turn initiate a cascade of cellular damage, including protein mutation, lipid peroxidation, enzyme inhibition, and DNA damage.

One of the primary mechanisms through which green nanoparticles exert antimicrobial effects is the generation of reactive oxygen species (ROS). ROS such as hydrogen peroxide (H_2_O_2_), superoxide anions (O^2−^), and hydroxyl radicals (OH^−^) can cause oxidative stress within microbial cells. This oxidative stress damages cellular components, including lipids, proteins, and DNA [209,210]. For instance, a study by Singhal et al. (2011) demonstrated that silver nanoparticles synthesized using *Ocimum sanctum* (holy basil) extract generated significant levels of ROS, leading to oxidative damage in *Escherichia coli* and *Staphylococcus aureus* cells. The ROS-induced damage resulted in membrane lipid peroxidation, protein denaturation, and DNA fragmentation, ultimately causing cell death [211] (Figure 5).

### 5.2. Interaction with Microbial Cell Membranes

Green nanoparticles can interact directly with microbial cell membranes, leading to physical disruption and loss of membrane integrity. This interaction is facilitated by the electrostatic attraction between the negatively charged microbial cell walls and the positively charged nanoparticles [212]. The nanoparticles adhere to the cell surface, causing structural changes and increased permeability. Once adhered, the nanoparticles insert themselves into the lipid bilayer of the cell membrane. This insertion is facilitated by the small size and high surface area of the nanoparticles, allowing them to embed within the lipid matrix. The embedded nanoparticles disrupt the lipid bilayer, creating pores and increasing membrane permeability, which leads to leakage of intracellular contents and loss of membrane potential [205]. For example, Bawazeer et al. (2022) reported that gold nanoparticles synthesized using *Piper nigrum* (black pepper) extract adhered to the cell membrane of *E. coli* and *Aspergillus flavus*, causing membrane deformation and leakage of intracellular contents. This disruption of the cell membrane compromised the integrity of the microbial cell, leading to cell lysis and death [213].

### 5.3. Penetration and Disruption of Microbial Cell Membranes

Green nanoparticles penetrate microbial cell membranes primarily through physical interactions that disrupt membrane integrity. The penetration process often involves several steps:

Adherence to the Cell Surface: The nanoparticles first adhere to the microbial cell surface due to electrostatic interactions [214].

Membrane Insertion: Once adhered, the nanoparticles insert themselves into the lipid bilayer of the cell membrane. This insertion is facilitated by the small size and high surface area of the nanoparticles, which allows them to embed within the lipid matrix [215].

Membrane Disruption: The embedded nanoparticles disrupt the lipid bilayer, creating pores and causing increased membrane permeability. This disruption leads to the leakage of cellular contents and loss of membrane potential [216].

**Figure 6:**
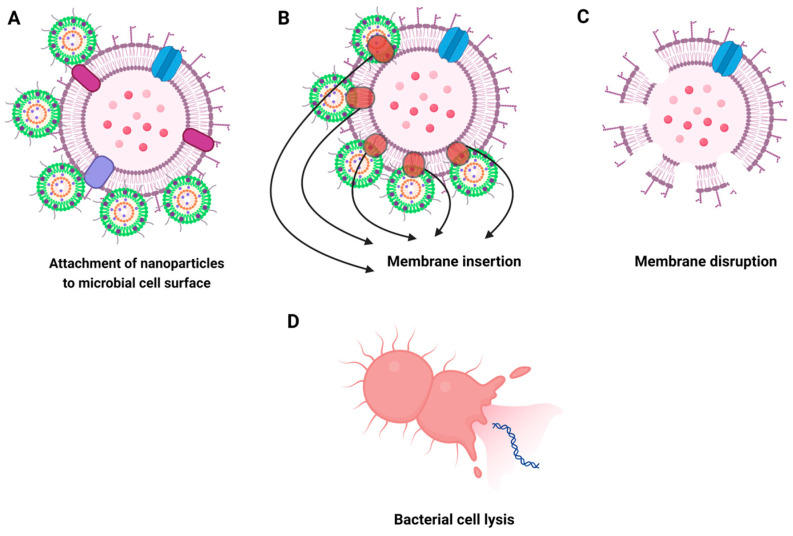
Mechanism of nanoparticle-induced bacterial cell lysis through membrane disruption. (**A**) Attachment of nanoparticles to microbial cell surface: Nanoparticles specifically attach to the microbial cell membrane, initiating the interaction process. (**B**) Membrane insertion: Following attachment, nanoparticles insert themselves into the microbial membrane, disrupting its integrity. (**C**) Membrane disruption: The insertion of nanoparticles leads to significant disruption of the microbial membrane, compromising its structure and function. (**D**) Bacterial cell lysis: As a result of membrane disruption, the bacterial cell undergoes lysis, releasing its internal contents and leading to cell death.

A study by Varghese et al. (2017) demonstrated that silver nanoparticles synthesized using *Trigonella foenum-graecum* (fenugreek) extract effectively disrupted the cell membranes of *Pseudomonas aeruginosa*. The nanoparticles caused significant membrane damage, resulting in the leakage of cytoplasmic contents and cell death [217] (Figure 6).

### 5.4. Interference with Intracellular Components

Green nanoparticles can penetrate microbial cells and interact with intracellular components, disrupting essential metabolic processes. Once inside the cell, nanoparticles can bind to enzymes and proteins, inhibiting their function and leading to metabolic dysregulation. Additionally, nanoparticles can interact with nucleic acids, preventing DNA replication and transcription [218]. A study by Otari et al. (2015) demonstrated the intracellular synthesis of silver nanoparticles (AgNPs) using the actinobacteria *Rhodococcus* spp. The study found that the synthesis of AgNPs occurred inside the bacterial cells, specifically in the cytoplasm. These nanoparticles showed excellent bactericidal and bacteriostatic activity against pathogenic microorganisms by interacting with intracellular components, disrupting essential metabolic processes, and leading to bacterial cell death [219].

### 5.5. Biofilm Inhibition

Microbial biofilms, which are structured communities of microbial cells enclosed in a self-produced polymeric matrix, pose significant challenges in clinical settings due to their resistance to conventional antibiotics. Green nanoparticles have been shown to inhibit biofilm formation and disrupt established biofilms. This anti-biofilm activity is attributed to the nanoparticles’ ability to penetrate the biofilm matrix and interact with biofilm-forming cells. For instance, a study by Swidan et al. (2022) demonstrated that silver nanoparticles synthesized using a green method showed significant antibiofilm activity against various bacterial strains. The nanoparticles inhibited quorum sensing and penetrated the biofilm matrix, leading to the disruption of biofilm structure and inhibition of biofilm formation [220].

These specific molecular interactions highlight the multifaceted antimicrobial mechanisms of green nanoparticles. Their ability to generate ROS, disrupt and penetrate cell membranes, interfere with intracellular components, and inhibit biofilm formation underscores their potential as effective antimicrobial agents [218]. For instance, NPs are responsible for the generation of ROS, and the radicals produced as a result of ROS generation are responsible for the oxidative stress induction and membrane lipid modification. This is accomplished by structural modification of mitochondrial DNA and cell proteins and may also result in lipid oxidation or mutagenesis (Figure 4). This alters the development of the inflammatory process, which may ultimately result in the death of cells or inflammation [221]. As a result, NPs are beneficial for combating harmful germs. Because they differ in their modes of action from conventional antibiotics, this is particularly interesting [33,222].

The antibacterial action of nanoparticles highly depends on their physicochemical characteristics. It should be noted, however, that the bacterial strain or the ambient circumstances are also highly significant for nanoparticle–microbial interaction [223,224]. It has previously been shown that the test medium’s temperature and pH affect the in vitro nanoparticle solubility and antibacterial activity [225]. The pH should be lowered since this improves the solubility of the NPs and amplifies their antibacterial action. Because of this, an acidic environment improved the solubility of nanoparticles [226,227]. The diffusion approach was shown to be appropriate for testing AgNPs owing to the release of highly diffusive and toxic ions into the culture medium. This was discovered when AgNPs and AuNPs were subjected to testing. Au ions are not discharged into the media, even though the antibacterial activity of AuNPs is caused by physical contact with the bacterium [228].

NPs can interact with bacterial cells, govern cell membrane penetration, and disrupt molecular pathways [218,229,230]. They can do this by acting as nanoscale molecules. The majority of nanoparticles, including AgNPs, have a low permeability barrier and may quickly enter cells. It is dependent on the kind of cell as to whether endocytosis or macropinocytosis is used for the absorption of AgNPs by non-transforming cells. Based on these considerations, the effect of NPs on the membrane of a bacterial cell is two-fold: first, there is a breakdown of the membrane potential and integrity, and second, ROS generation occurs. Other processes that are now recognized include stimulating host immunological responses, inhibiting biofilm development, and inhibiting protein and RNA synthesis via intracellular effects. This approach also inhibits biofilm formation [214,231]. The treatment of infections in the anterior segment of the eye using ocular medication delivery systems available on the market is ineffective. NPs were explicitly developed for use in manufacturing preparations for injectable solutions and ocular drops. Janagam et al. found that medications loaded with NPs had improved pharmacodynamics, pharmacokinetics, immunogenicity, non-specific toxicity, and biorecognition, which resulted in a surge in the overall effectiveness of the treatments [232]. A practical method for multifunctional polyelectrolyte thin film production in the loading and distribution of medicinal medicines was disclosed by Sripriya et al., wherein AgNPs that were biologically synthesized from *Hybanthus enneaspermus* leaf extract were identified to be useful in the role of reducing agents with substantial promise for antibacterial coatings, wound dressings and slightly triggered drug delivery [233,234].

## 6. Safety and Biocompatibility of Green-Synthesized Nanoparticles

Green nanoparticles, synthesized using environmentally friendly methods involving biological entities such as plant extracts, bacteria, fungi, and algae, are increasingly recognized for their safety and biocompatibility compared to regular nanoparticles produced through conventional chemical methods. The primary advantage of green nanoparticles lies in their synthesis process, which eliminates the need for toxic chemicals and harsh reducing agents. This eco-friendly approach not only reduces environmental impact but also enhances the biocompatibility of the nanoparticles, making them safer for use in biomedical applications.

Several studies have highlighted the improved safety profile of green nanoparticles. For instance, Mani et al. (2023) conducted a comparative study on the anti-cancer and anti-oxidant effects of aqueous plant leaf extract alone and AgNPs synthesized using phytoconstituents of *Eclipta alba* leaf extract on human triple-negative breast cancer cells (MDA-MB-231). The study found that the green-synthesized AgNPs exhibited 2.6-fold higher anti-oxidant potential showing dose-dependent cytotoxicity to induce apoptosis compared to the leaf extract alone [235]. Another study by Khan and Javed (2021) examined the differential impact of chemically synthesized silver nanoparticles (AgNPs) and green AgNPs synthesized using *Azadirachta indica*. The green-synthesized AgNPs demonstrated maximum antimicrobial activity against both *E. coli* and *S. aureus* while showing no antimicrobial activity by the leaf extract alone. The natural phytochemicals present in the plant extract played a crucial role in inhibition of bacterial growth due to greater occupancy of phytochemicals on the particle surface that improved their binding affinity towards bacterial cell membrane or the excessive Ag^+^ release [236]. In addition to their safety on human cells, green nanoparticles have been shown to have a less disruptive impact on the human microbiota. A study conducted by Bidian et al. (2023) demonstrated that chemically synthesized gold and silver nanoparticles were toxic to stem cells, while green-synthesized nanoparticles did not exhibit such toxicity, suggesting a potentially safer profile for gut microbiota as well [237]. A study by Alsaiari et al. (2023) supported the eco-friendly nature and non-toxicity of green-synthesized nanoparticles, further implying their benign impact on biological systems including gut microbiota [238].

Similarly, a study by Ishwarya et al. (2018) explored the influence of green-synthesized zinc oxide nanoparticles using *Ulva lactuca* seaweed extract for its antibacterial and larvicidal activity. Ul-ZnO nanoparticles (NPs) demonstrated excellent bactericidal activity against both Gram-positive bacteria (*Bacillus licheniformis* and *Bacillus pumilis*) and Gram-negative bacteria (*Escherichia coli* and *Proteus vulgaris*). They also exhibited high antibiofilm potential in both dark and sunlight conditions. Additionally, Ul-ZnO NPs achieved 100% mortality of *Aedes aegypti* 4th instar larvae at a concentration of 50 μg/mL within 24 h. These findings suggest that synthesizing multifunctional Ul-ZnO NPs from widely available seaweed products can be a potential eco-friendly alternative to chemical methods currently used for producing antimicrobials and insecticides [239]. The enhanced safety profile of green nanoparticles, as demonstrated by these studies, underscores their potential as a sustainable and biocompatible alternative to conventional nanoparticles. By avoiding the use of toxic chemicals in their synthesis, green nanoparticles offer a safer option for various biomedical and environmental applications.

The long-term effects of green nanoparticle exposure on human cells and tissues are a critical aspect of their safety profile, especially given their potential applications in medicine and consumer products. Although green nanoparticles are generally considered safer and more biocompatible than their chemically synthesized counterparts, understanding their chronic effects is essential for assessing their overall safety.

### 6.1. Cytotoxicity and Genotoxicity

One of the key concerns regarding long-term exposure to green nanoparticles is their potential for cytotoxic and genotoxic effects. Studies have shown that while green nanoparticles are less cytotoxic compared to conventional nanoparticles, prolonged exposure can still result in cellular stress and damage. For instance, a study by Khor et al. (2020) evaluated the cytotoxic effects of silver nanoparticles (AgNPs) synthesized using *Moringa oleifera* leaf extract on Kasumi-1 leukemia cell line. The study found that after prolonged exposure, there was a significant increase in reactive oxygen species (ROS) production and DNA damage, indicating potential genotoxic effects. However, the effects were significantly lower than those observed with chemically synthesized AgNPs [240]. Green nanoparticles, due to their biocompatible synthesis methods, generally exhibit lower cytotoxicity and genotoxicity compared to chemically synthesized counterparts. For example, a study by Dowlath et al. (2021) demonstrated that green-synthesized ferrous oxide nanoparticles showed significantly reduced cytotoxic effects on human peripheral blood mononuclear cells compared to chemically synthesized nanoparticles, which caused notable DNA damage and cell viability reduction, while reporting superior antibacterial activity [241].

In another case, one study demonstrated that green-synthesized CS/SiO_2_/TiO_2_/CeO_2_/Fe_3_O_4_ nanocomposites exhibit superior biocompatibility, anti-inflammatory, and antibacterial properties compared to chemically synthesized counterparts. Green synthesis using natural extracts provides an environmentally friendly and cost-effective alternative, resulting in nanocomposites with reduced cytotoxicity in vitro and in vivo. The enhanced biological properties and lower toxicity of green-synthesized nanocomposites make them more suitable for biomedical applications [242].

However, despite these promising findings, additional long-term in vivo studies and clinical data are crucial for comprehensively understanding the long-term safety and efficacy of green nanoparticles. Further studies examining the chronic exposure effects, biodistribution, and potential accumulation of green nanoparticles in various tissues will provide valuable insights into their long-term biocompatibility and potential risks. Comparative analyses with chemically synthesized nanoparticles in these studies will further validate the advantages of green synthesis methods in reducing adverse health impacts.

### 6.2. Inflammatory Responses

Another aspect of long-term nanoparticle exposure is the potential to induce chronic inflammatory responses. Chronic inflammation can lead to various diseases, including cancer and fibrosis. A study by Nagajyothi et al. (2015) investigated the long-term inflammatory effects of zinc oxide nanoparticles (ZnO NPs) synthesized using *Polygala tenuifolia* root extract. The results showed that after prolonged exposure, the green-synthesized ZnO NPs caused a significant suppression in the expression of inflammatory cytokines such as IL-6 and TNF-α in human cells. However, this suppression is in contrast to that induced by chemically synthesized ZnO NPs, suggesting a more favorable inflammatory profile for green-synthesized nanoparticles [243].

### 6.3. Effects on Human Microbiota

One of the key concerns regarding the long-term exposure to green nanoparticles is their potential for cytotoxic and genotoxic effects. Studies have shown that while green nanoparticles are less cytotoxic compared to conventional nanoparticles, prolonged exposure can still result in cellular stress and damage. For instance, a study by Ullah et al. (2020) evaluated the long-term cytotoxic effects of silver nanoparticles (AgNPs) synthesized using *Fagonia indica* extract on human breast cancer cells (MCF-7). The study found that after prolonged exposure, there was a significant increase in reactive oxygen species (ROS) production and DNA damage, indicating potential genotoxic effects. However, the effects were notably lower than those observed with chemically synthesized AgNPs, suggesting a more favorable profile for green-synthesized nanoparticles [244].

Several studies therefore highlight the relatively lower long-term toxicity of green nanoparticles compared to their chemically synthesized counterparts. However, it is crucial to continue long-term studies and toxicological assessments to fully understand the safety profile of green nanoparticles, especially as their use in various applications continues to expand.

The human microbiome, comprising trillions of microorganisms residing in various body sites, plays a crucial role in maintaining health and homeostasis. Disruptions to the microbiome composition can lead to various health issues, including gastrointestinal disorders, metabolic diseases, and infections. Green nanoparticles, synthesized using biological entities such as plant extracts, offer a potentially less disruptive alternative to chemically synthesized nanoparticles.

#### 6.3.1. Gut Microbiome

The impact of long-term exposure to green-synthesized nanoparticles on the human microbiota, particularly the gut microbiota, is an area of growing interest. The gut microbiota plays a crucial role in maintaining overall health, and disruptions to its composition can lead to various health issues. Green nanoparticles, due to their biocompatible synthesis methods, generally exhibit lower cytotoxicity and genotoxicity compared to chemically synthesized counterparts. They have shown a potentially safer profile compared to chemically synthesized nanoparticles, particularly concerning their impact on the human microbiome. Recent studies indicate that green nanoparticles, due to their eco-friendly synthesis methods, exhibit lower toxicity towards beneficial gut bacteria. Several studies show that titanium dioxide (TiO_2_) nanoparticles generally have minimal effects on the gut microbiome at low concentrations. For instance, a study using a model intestinal bacterial community and doses equivalent to consuming 1–2 pieces of gum found only minor changes, such as reduced *Bacteroides ovatus* and increased *Clostridium cocleatum* [245]. Similarly, an in vitro Human Gut Simulator and a mouse study with a dose of 2.5 mg/kg bw/day for 7 days found no significant changes in microbial composition, diversity, or functionality despite a slight reduction in community density [246].

Similarly, another study by Pereira et al. (2014) highlighted that green-synthesized iron (III) oxo-hydroxide nanoparticles were less disruptive to the human gut microbiome, preserving the balance of beneficial bacteria (*Lactobacillus*) [247]. These studies suggest that green nanoparticles could offer a safer alternative for applications involving direct human exposure, such as in medical treatments and food packaging.

A study by Wilding et al. (2015) explored the effects of silver nanoparticles (AgNPs) synthesized using green methods on gut microbiota. The study found that repeated long-term exposure to these green-synthesized AgNPs did not significantly alter the gut microbiota composition, preserving the balance of beneficial bacteria [248]. In contrast, chemically synthesized AgNPs caused a reduction in microbial diversity and a shift towards a more pathogenic microbiota profile [249]. Another study by Morais et al. (2020) investigated the cytotoxic effects of green-synthesized silver nanoparticles on different bacterial strains. The results indicated that green-synthesized AgNPs maintained the health of beneficial gut bacteria while effectively targeting pathogenic strains. Chemically synthesized AgNPs, however, were less selective and caused broader disruptions in gut microbiota composition [250]. These studies suggest that green-synthesized nanoparticles are less likely to disturb the delicate balance of the gut microbiota compared to chemically synthesized nanoparticles. However, more detailed studies on the functional aspects of the microbiome and the long-term implications of exposure to green nanoparticles are necessary. These comprehensive studies will help elucidate the long-term safety profile of green nanoparticles and their suitability for widespread use.

#### 6.3.2. Skin Microbiome

The skin microbiome, consisting of diverse microbial communities, is essential for skin health and protection against pathogens. A study by Rani et al. (2022) evaluated the effects of green-synthesized zinc oxide nanoparticles (ZnO NPs) on the skin microbiota. The nanoparticles were synthesized using *Hibiscus sabdariffa* leaf extract and applied topically to human skin. The study found that the green-synthesized ZnO NPs did not significantly disrupt the composition or function of the skin microbiota, suggesting their suitability for topical applications. In contrast, chemically synthesized ZnO NPs were found to disrupt the skin microbiota, reducing the abundance of beneficial microbes and potentially increasing the risk of skin infections [251]. Another study by Faisal et al. (2021) investigated the effects of green-synthesized zinc oxide nanoparticles using *Myristica fragrans* (nutmeg) fruit extract. The results showed that the green-synthesized ZnO NPs preserved the balance of beneficial microbes and maintained the overall diversity of the skin microbiota. In contrast, chemically synthesized ZnO NPs led to a reduction in microbial diversity and increased the risk of pathogenic infections [252].

#### 6.3.3. Oral Microbiome

The oral microbiome plays a vital role in oral health, influencing dental and periodontal diseases. A study by Salman et al. (2022) investigated the effects of green-synthesized silver nanoparticles on the oral microbiota. The nanoparticles were synthesized using *Rhus coriaria* (sumac) extract and tested for their impact on oral pathogenic microorganisms [253]. The results showed that green-synthesized AgNPs had minimal effects on the overall composition of the oral microbiota, preserving beneficial bacteria while inhibiting pathogenic species. This contrasts with chemically synthesized AgNPs, which were found to disrupt the balance of the oral microbiota, leading to an increase in pathogenic bacteria.

Green nanoparticles exhibit distinct pharmacokinetic and pharmacodynamic behaviors in in vivo models, shaped by their biocompatible and eco-friendly synthesis methods. Understanding these unique characteristics is essential for comprehensively assessing their safety and potential in biomedical applications.

### 6.4. Pharmacokinetics of Green Nanoparticles

Absorption and Bioavailability: Green nanoparticles often show enhanced bioavailability due to their natural capping agents. A study by Premanand et al. (2016) demonstrated that green-synthesized silver nanoparticles using *Nelumbo nucifera* extract had higher bioavailability and faster absorption rates compared to chemically synthesized counterparts. The presence of phytochemicals from the plant extract improved nanoparticle solubility and stability in the gastrointestinal tract [254].

Distribution: These nanoparticles typically accumulate in organs like the liver, spleen, kidneys, and lungs due to the enhanced permeability and retention (EPR) effect. Ibrahim et al. (2018) showed that green-synthesized gold nanoparticles were predominantly distributed to the liver and spleen in mice, indicating their potential for targeted delivery [255].

Metabolism: Green nanoparticles are metabolized primarily in the liver. Natural capping agents enhance their metabolic stability, reducing toxicity. Ali et al. (2024) found that zinc oxide nanoparticles synthesized using *egg albumin* were metabolized in the liver, with metabolites excreted through bile and urine, thus enhancing metabolic stability and reducing toxicity [256].

Excretion: Green nanoparticles are excreted via renal and hepatic pathways. Patel et al. (2016) observed that copper nanoparticles synthesized using *Ocimum sanctum* extract were primarily excreted through urine, demonstrating efficient renal clearance [257].

### 6.5. Pharmacodynamics of Green Nanoparticles

Mechanism of Action: The pharmacodynamic effects of green nanoparticles are mediated through their antimicrobial, anti-inflammatory, and anticancer properties. Masurkar et al. (2011) demonstrated that silver nanoparticles synthesized using *Cymbopogon citratus* (lemongrass) extract exhibited strong antibacterial activity against *Staphylococcus aureus* and *Escherichia coli*. The mechanism involved the generation of reactive oxygen species (ROS), disruption of bacterial cell membranes, and inhibition of DNA replication [258].

Therapeutic Efficacy: The therapeutic efficacy of green nanoparticles has been demonstrated in various models. Siddiq et al. (2019) evaluated the anticancer efficacy of gold nanoparticles synthesized using *Azadirachta indica* (neem) extract in vitro model of breast cancer. The results showed significant inhibition of tumor growth and induction of apoptosis in cancer cells. The natural capping agents from neem extract enhanced therapeutic efficacy by facilitating targeted delivery and reducing systemic toxicity [259].

Safety and Toxicity: Green-synthesized nanoparticles generally exhibit lower toxicity compared to chemically synthesized nanoparticles due to biocompatible capping agents. Packialakshmi et al. (2021) found that zinc oxide nanoparticles synthesized using *Mentha piperita* (peppermint) extract caused minimal toxicity to the liver and kidneys, with no significant changes in hematological and biochemical parameters. Natural capping agents from peppermint extract reduced oxidative stress and inflammation [260].

These studies indicate that green nanoparticles, due to their biocompatible and eco-friendly synthesis methods, are less likely to disrupt the human microbiome compared to conventional nanoparticles. However, further long-term studies and comprehensive assessments are necessary to fully understand the impact of green nanoparticles on different microbial communities within the human body.

## 7. Current Clinical Scenario for Green Nanoparticles

Recent advancements in green nanoparticle research have highlighted their potential in treating phytopathogens, with several case studies and clinical trials demonstrating their efficacy. For instance, a clinical trial conducted by You et al. (2017) evaluated the use of green-synthesized silver nanoparticles in promoting wound healing [261]. Khan et al. (2021) found that silver nanoparticles synthesized using *Azadirachta indica* (Neem) leaf extract showed significant reduction in bacterial load against *Escherichia coli* (Gram −ve) and *Staphylococcus aureus* (Gram +ve) compared to conventional treatments. The effectiveness of these nanoparticles was attributed to their ability to generate reactive oxygen species (ROS) and disrupt bacterial cell membranes, leading to effective bacterial clearance [236].

In another clinical trial, Asker et al. (2024) investigated the application of gold nanoparticles synthesized using *Pelargonium graveolens* leaf extract in treating multidrug-resistant infections. This study focused on patients with infections caused by *Streptococcus mutans* and *Candida albicans*. The gold nanoparticles exhibited excellent antimicrobial activity, disrupting bacterial cell walls and inducing oxidative stress within the cells [200]. Patients treated with these nanoparticles experienced faster recovery times and fewer side effects compared to those receiving standard antibiotic therapy, demonstrating the potential of green nanoparticles in addressing antibiotic resistance.

Combining green nanoparticles with conventional antibiotics can further enhance the efficacy of antimicrobial treatments, offering a promising strategy to combat antibiotic resistance and improve therapeutic outcomes. Several studies have explored the synergistic effects of such combinations, demonstrating improved antimicrobial activity against various pathogens.

### 7.1. Silver Nanoparticles and Antibiotics

Silver nanoparticles (AgNPs) synthesized using green methods have shown significant potential when combined with conventional antibiotics. For instance, a study by Fayaz et al. (2010) investigated the synergistic effects of green-synthesized AgNPs using *Tridax procumbens* leaf extract with antibiotics like ampicillin and kanamycin against *Staphylococcus aureus* and *Escherichia coli*. The results demonstrated enhanced antibacterial activity, with lower minimum inhibitory concentrations (MICs) required for the combinations compared to the antibiotics alone. The mechanism involved AgNPs disrupting bacterial cell membranes, allowing increased antibiotic penetration and effectiveness [262].

Another study by Gurunathan et al. (2014) evaluated the combination of green-synthesized AgNPs using *Pseudomonas aeruginosa* with tetracycline against *Klebsiella pneumoniae*. The study found that the combination exhibited a synergistic effect, significantly reducing bacterial viability. The green-synthesized AgNPs enhanced the antibiotic’s ability to penetrate biofilms and target bacterial cells, leading to improved treatment outcomes [263].

### 7.2. Gold Nanoparticles and Antibiotics

Gold nanoparticles (AuNPs) synthesized using plant extracts have also shown promising results in combination with antibiotics. For instance, a study focused on the potential antibacterial applications of green-synthesized AuNPs. Bharadwaj et al. (2021) provided an overview of green-synthesized AuNPs, including their characterization methods and applications in cancer therapy. However, they also highlighted the nanoparticles’ significant antibacterial potential when used in conjunction with traditional antibiotics. This study emphasized that the combination of green-synthesized AuNPs with conventional antibiotics could be a viable strategy to combat antibiotic-resistant bacterial infections [264].

### 7.3. Copper Nanoparticles and Antibiotics

Copper nanoparticles (CuNPs) also show great potential when used in conjunction with conventional antibiotics. A study by Woźniak-Budych et al. (2017) investigated the antimicrobial properties of copper nanoparticles loaded with rifampicin. The study demonstrated that the combination of CuNPs and rifampicin exhibited significant antibacterial activity against several strains, including *Staphylococcus aureus* and *Pseudomonas aeruginosa*. The synergistic effect was attributed to the nanoparticles’ ability to enhance the antibiotic’s penetration into bacterial cells, thereby increasing its efficacy [265].

Many studies have discussed the potential of copper nanoparticles as alternatives to conventional antibiotics. They have emphasized the nanoparticles’ effectiveness against various bacterial, fungal, and viral infections due to their unique mechanisms of action, such as disrupting bacterial cell membranes and generating reactive oxygen species (ROS). The combination of CuNPs with antibiotics could thus offer a powerful strategy to address the growing issue of antibiotic resistance [266].

These studies highlight the potential of green nanoparticles to enhance the efficacy of conventional antibiotics through synergistic interactions. The optimal combinations vary depending on the type of nanoparticle, the antibiotic, and the target pathogen. Understanding these interactions can lead to the development of more effective antimicrobial therapies, particularly in the fight against antibiotic-resistant infections.

## 8. Challenges Associated with Clinical Translation and Their Prospective Solutions

Despite the promising results from these case studies and clinical trials, several challenges remain in translating green nanoparticle research into clinical practice. Scaling up the production of green nanoparticles while maintaining consistency and quality is a significant hurdle [267]. The variability in biological sources and extraction methods can lead to differences in nanoparticle size, shape, and efficacy [268]. Standardized protocols for nanoparticle synthesis are essential to overcome this issue. Additionally, regulatory approval for green nanoparticles requires rigorous testing to meet safety and efficacy standards, necessitating extensive in vitro and in vivo studies to assess their toxicological profiles and therapeutic potential [152]. Ensuring the stability of green nanoparticles during storage and transportation is crucial for their clinical application, with encapsulation techniques and stabilizing agents playing a key role in enhancing their shelf-life [269]. Furthermore, the cost of producing green nanoparticles must be competitive with conventional treatments to encourage widespread adoption. Optimizing synthesis methods to reduce costs without compromising quality, and utilizing waste biomass and renewable plant resources, can contribute to cost reduction. Addressing these challenges will facilitate the effective translation of green nanoparticles from laboratory research to clinical settings, offering a sustainable and biocompatible alternative to conventional antimicrobials.

Scaling up the synthesis of green nanoparticles for industrial production while maintaining eco-friendliness and cost-effectiveness involves several critical considerations. Achieving this requires optimizing various aspects of the production process, from the selection of raw materials to the implementation of advanced manufacturing technologies.

One effective strategy is the use of continuous flow reactors, which offer several advantages over traditional batch synthesis methods. Continuous flow reactors enable precise control over reaction parameters such as temperature, pressure, and flow rates, leading to consistent and high-quality nanoparticle production. One example of scaling up green-synthesized nanoparticles in continuous flow reactors is demonstrated in the study by I. Assaf et al. (2023). This study designed a scalable tubular flow reactor for the continuous production of stable alkali lignin nanoparticles. The reactor successfully maintained consistent nanoparticle production, which indicates the feasibility of scaling up green synthesis methods using continuous flow systems [270].

Additionally, J. Mahin et al. (2021) demonstrated the continuous, green, scalable, and reproducible synthesis of PEG-functionalized iron nanoparticles using a flow synthesis method. This approach highlights the advantages of continuous flow reactors in achieving high stability and uniformity in nanoparticle production while being cost-effective and environmentally friendly [271]. Continuous flow reactors have been successfully implemented in several industrial settings to enhance the scalability of green nanoparticle synthesis. These reactors offer precise control over reaction conditions, consistent nanoparticle quality, and reduced batch-to-batch variations. However, challenges such as initial capital investment, specialized equipment, and the need for trained personnel can pose barriers to widespread adoption, particularly for small- and medium-sized enterprises. Addressing these challenges through government incentives, industry partnerships, and ongoing R&D is crucial to facilitating broader adoption.

Maintaining eco-friendliness and cost-effectiveness in the scale-up of green nanoparticle synthesis involves utilizing renewable plant-based materials, optimizing reaction conditions to minimize energy use, and integrating waste management practices. The green synthesis of nanomaterials using waste materials is an emerging research area focusing on sustainable methods to produce desirable products with minimal waste [272]. To further enhance the eco-friendliness and cost-effectiveness of the synthesis process, the use of waste biomass as a raw material can be considered. Utilizing agricultural or industrial waste not only reduces the cost of raw materials but also contributes to waste valorization [273,274]. A study by Chaudhuri et al. (2017) demonstrated the synthesis of zinc oxide nanoparticles using waste tea leaves extract. The process not only provided a cost-effective raw material source but also aligned with sustainable waste management practices [275]. Various wastes, such as agricultural and fruit residues, have been used successfully to synthesize silver nanoparticles (AgNPs), leveraging biomolecules like starches, proteins, and phenols [276]. This approach offers significant economic benefits and has seen increasing interest in recent years. Examples include the use of banana peel, papaya peel, tea wastes, sugarcane bagasse, orange peel, cauliflower waste, and *Eucalyptus camadulensis* bark [277]. The adoption of green chemistry principles, such as solvent-free reactions and non-toxic solvents, reducing energy consumption, and optimizing reaction conditions to maximize efficiency and yield, further enhances eco-friendliness. For instance, optimizing the concentration of biological extracts and metal salts can enhance the reaction efficiency and reduce the amount of raw materials required, leading to cost savings and reduced environmental impact [152]. Companies face challenges like maintaining product consistency, managing supply chains for raw materials, and meeting regulatory standards. Solutions include implementing stringent quality control measures, establishing reliable supplier networks, and engaging with regulatory bodies to ensure compliance with safety and environmental standards. These strategies ensure that the scalability of green nanoparticle synthesis can be achieved, paving the way for widespread adoption of eco-friendly and cost-effective nanotechnology solutions.

Furthermore, collaboration between academic researchers, industry stakeholders, and regulatory agencies is crucial for the successful scaling up of green nanoparticle synthesis. Establishing standardized protocols for large-scale production and conducting thorough environmental and economic impact assessments can facilitate the adoption of green nanoparticles in various industrial applications.

## 9. Standardization, Regulation and Environmental Effects of Green Nanoparticles

### 9.1. Standardization

Standardized protocols for characterizing green nanoparticles are essential for ensuring consistency, reproducibility, and reliability of research findings. These protocols should encompass various stages of nanoparticle synthesis, characterization, and application. Developing and implementing these protocols involves a systematic approach that integrates standardized methods, advanced characterization techniques, and comprehensive case studies.

The first step in standardizing green nanoparticle characterization is to develop uniform synthesis protocols. This involves the selection of consistent biological reducing agents, such as using plant extracts or microbial sources as reducing and stabilizing agents. For instance, a study by Nkosi et al. (2024) highlighted the use of *Ocimum sanctum* (holy basil) for the synthesis of silver nanoparticles, providing a standardized approach to biological synthesis [278]. Maintaining controlled synthesis conditions, such as consistent temperature, pH, and concentration of reactants, ensures reproducibility. Singh et al. (2023) provided a systematic approach for nanoparticle synthesis using *Azadirachta indica* (neem), ensuring controlled and reproducible conditions [279].

Characterization of green nanoparticles involves several advanced techniques to determine their physical, chemical, and biological properties. Standard characterization techniques include ultraviolet–visible (UV–Vis) spectrophotometry for initial confirmation of nanoparticle synthesis by analyzing surface plasmon resonance (SPR) peaks. Alharbi et al. (2022) demonstrated the use of UV–Vis spectrophotometry for characterizing silver nanoparticles synthesized using *Ficus carica* (fig) extract [280]. Fourier transform infrared (FTIR) spectroscopy identifies functional groups on nanoparticle surfaces, indicating the presence of stabilizing agents. Ali et al. (2023) used FTIR to characterize silver nanoparticles synthesized with garlic extract, ensuring the presence of organic capping agents [281]. Transmission electron microscopy (TEM) and scanning electron microscopy (SEM) provide detailed images of nanoparticle size, shape, and morphology. Osman et al. (2024) employed TEM and SEM for characterizing green-synthesized gold nanoparticles, revealing their uniform size and spherical shape [152]. X-ray diffraction (XRD) determines the crystalline structure of nanoparticles. Ying et al. (2022) used XRD to analyze the crystalline nature of silver nanoparticles synthesized using *Camellia sinensis* (green tea) extract [150].

Implementing standardized protocols involves validating them through comprehensive case studies. For example, a study by Nkosi et al. (2024) used a standardized synthesis method with *Ocimum sanctum* extract and characterized the nanoparticles using UV–Vis, FTIR, TEM, and XRD, ensuring reproducibility and consistency in the properties of the synthesized nanoparticles [278]. Similarly, Dudhane et al. (2019) synthesized gold nanoparticles using *Terminalia arjuna* bark extract and characterized them using SEM, TEM, and XRD, demonstrating the feasibility of implementing standardized protocols across different nanoparticle types [282]. Developing standardized protocols also requires regulatory and collaborative efforts, including establishing guidelines that outline the required characterization techniques and reporting standards, and encouraging collaboration between research institutions, industry stakeholders, and regulatory bodies to develop and refine these protocols. Standardized protocols for characterizing green nanoparticles can enhance the reproducibility and reliability of research, facilitating their safe and effective application in various fields.

### 9.2. Environmental Effects

The large-scale production and use of green nanoparticles, despite their eco-friendly synthesis methods, can have several potential environmental consequences. These include impacts on soil, water, and air quality, as well as effects on plant and animal life.

#### 9.2.1. Soil Quality and Microbial Communities

Green nanoparticles, if released into the soil, can interact with soil microbes and alter the microbial community structure. These interactions may disrupt essential soil processes such as nutrient cycling and organic matter decomposition. Studies have shown that nanoparticles can affect the growth and function of beneficial soil bacteria and fungi, potentially leading to reduced soil fertility and health [150].

#### 9.2.2. Water Quality and Aquatic Life

The release of green nanoparticles into water bodies can lead to the contamination of aquatic ecosystems. Nanoparticles can adsorb pollutants and heavy metals, altering their bioavailability and toxicity. Furthermore, nanoparticles can directly affect aquatic organisms. For instance, silver nanoparticles are known to be toxic to various aquatic species, including fish and invertebrates, causing oxidative stress and cellular damage. These impacts can disrupt aquatic food webs and reduce biodiversity [278].

#### 9.2.3. Air Quality and Human Health

During the production and application of green nanoparticles, airborne nanoparticles can be released into the atmosphere. Inhalation of these nanoparticles poses health risks to humans, including respiratory and cardiovascular issues. Additionally, nanoparticles in the air can contribute to air pollution and may have indirect effects on climate by influencing atmospheric processes [279].

#### 9.2.4. Bioaccumulation and Biomagnification

Green nanoparticles can enter food chains through bioaccumulation in plants and animals. Once in the food chain, these nanoparticles can biomagnify, leading to higher concentrations in top predators, including humans. This can pose health risks and affect the health of wildlife populations [281].

#### 9.2.5. Ecotoxicological Effects

The ecotoxicological effects of green nanoparticles are influenced by their size, shape, concentration, and surface chemistry. Studies have demonstrated that even low concentrations of nanoparticles can have significant toxic effects on various organisms. Long-term exposure can lead to chronic health issues in wildlife, including reproductive and developmental problems [280].

Mitigating these potential environmental consequences requires the development of safe disposal and containment strategies, comprehensive risk assessments, and stringent regulations governing the production and use of green nanoparticles. Continuous monitoring and research are essential to understand and manage the environmental impacts of green nanoparticles effectively.

### 9.3. Sustainable Practices in Synthesis

Integrating sustainable practices into the lifecycle of green nanoparticles, from synthesis to disposal, involves adopting eco-friendly methods, ensuring efficient resource use, and minimizing environmental impacts. This comprehensive approach can be achieved through the following strategies.

#### 9.3.1. Sustainable Synthesis Methods

The synthesis of green nanoparticles should prioritize the use of renewable resources and non-toxic materials. Utilizing plant extracts, microorganisms, and other biological entities as reducing and stabilizing agents can significantly reduce the environmental footprint. For instance, a study by Osman et al. (2024) highlighted the use of *Azadirachta indica* (neem) extract for the synthesis of silver nanoparticles, demonstrating a sustainable and eco-friendly approach [152]. Similarly, Oliveira et al. (2023) emphasized that green synthesis methods align with multiple sustainable development goals (SDGs), promoting environmental sustainability and reducing reliance on hazardous chemicals [283].

#### 9.3.2. Efficient Resource Utilization

Optimizing the use of raw materials and energy during nanoparticle synthesis can enhance sustainability. Implementing green chemistry principles, such as atom economy and energy efficiency, can minimize waste and reduce energy consumption. Wahab et al. (2024) discussed the benefits of using eco-friendly nanomaterial synthesis methods that require less energy and generate minimal waste, thereby promoting resource efficiency [284].

#### 9.3.3. Lifecycle Assessment (LCA)

Conducting lifecycle assessments (LCAs) from raw material extraction to nanoparticle disposal can identify potential environmental impacts and areas for improvement. LCA helps in evaluating the environmental performance of green nanoparticles throughout their lifecycle. A study by Dhingra (2010) highlighted the importance of incorporating LCA in the early stages of nanoparticle development to ensure sustainable practices are integrated from the beginning [285]. This approach enables the identification and mitigation of adverse environmental effects associated with nanoparticle production and use.

#### 9.3.4. Safe Disposal and Recycling

Developing safe disposal methods and recycling strategies for green nanoparticles is crucial to prevent environmental contamination. Encouraging the recovery and reuse of nanoparticles from waste streams can reduce the environmental burden. For example, recycling processes for silver nanoparticles can reclaim valuable metals and minimize waste. Additionally, promoting the biodegradation of green nanoparticles through natural processes can ensure their safe disposal without harming the environment [286].

#### 9.3.5. Regulatory Framework and Collaboration

Establishing stringent regulations and standards for the production, use, and disposal of green nanoparticles can ensure adherence to sustainable practices [152]. Collaboration between research institutions, industry stakeholders, and regulatory bodies is essential to develop and implement these standards. Regulatory frameworks can provide guidelines for safe manufacturing processes, proper waste management, and environmental monitoring.

#### 9.3.6. Case Studies and Implementation

Several case studies illustrate the successful integration of sustainable practices in green nanoparticle lifecycles. For instance, Dhingra’s (2010) study on sustainable nanotechnology emphasized the need for life cycle thinking in nanoparticle synthesis and highlighted practical examples of green synthesis methods that align with sustainability goals [285]. Another case study by Osman et al. (2024) demonstrated the application of green-synthesized nanoparticles in environmental remediation, showcasing their potential for sustainable use in various fields [152].

By adopting these strategies, the lifecycle of green nanoparticles can be made more sustainable, ensuring that their benefits do not come at the expense of environmental health.

## 10. Conclusions

The idea that plant pathogens may be responsible for diseases in animals and humans is unique and creates issues regarding the possibility of such infections in healthy individuals as well as people who are immunocompromised. Cross-kingdom infections are well-studied in nature, agriculture, hospitals, and households, but their prevalence in the general population is unknown. Humans ingest and digest plant tissues regularly, and the stomach’s minimal pH protects against hazardous plant-associated bacteria. Most human illnesses come from the respiratory tract, injured skin or people who are immunocompromised. Most cross-kingdom infections by phytopathogens are opportunistic, requiring no specific infection machinery. Active immunological disruption during macrophage phagocytosis by *P. syringae* shows opportunistic pathogens may establish cross-kingdom infection methods. Analysis of analogous mechanisms in animal and plant microbial pathogenesis in cross-kingdom hosts can assist in analyzing and characterizing rare diseases. 

Combining NPs so they may behave as nanobots has an exciting future: their interaction would vary non-linearly with factors like pH or temperature. This technique appears to be successful, and the prospect of nanotechnology is one step closer. Plant extract-mediated NP synthesis is ecologically friendly. The production of NPs from various plant extracts is a trend that might help characterize their method of action. It is an easily scalable controlled synthesis that assures biocompatibility and reduces environmental impact. Plant-based NPs have several medical and pharmacological uses. Studies demonstrate that NPs may exhibit anti-microbial effects alone or in conjunction with antibiotics, reducing antibiotic resistance due to overuse. Future studies should investigate the safety of plant-based NPs (environmental safety and that of human health) and reduce their environmental effect. More comprehensive production processes and the investigation of action mechanisms that underlie the anti-microbial impact of NPs are needed to make plant-based NPs a practical treatment approach.
